# Japanese participant data from three gantenerumab trials in early Alzheimer's disease

**DOI:** 10.1002/alz.70192

**Published:** 2025-04-29

**Authors:** Takashi Asada, Angeliki Thanasopoulou, Paul Delmar, Jakub Wojtowicz, Janice Smith, Yasumasa Yoshiyama, Katsunori Yokoi, Chigusa Watanabe, Mitsuhiro Isozaki, Ryoto Ozaki, Takaaki Ishida, Hironori Tatsuda, Akira Tamaoka

**Affiliations:** ^1^ Institute of Science Tokyo Tokyo Japan; ^2^ Roche Product Development Neuroscience, F. Hoffmann‐La Roche Ltd Basel Switzerland; ^3^ Product Development Roche Products Ltd Welwyn Garden City UK; ^4^ Inage Neurology and Memory Clinic Chiba Japan; ^5^ Department of Neurology National Center for Geriatrics and Gerontology Obu Japan; ^6^ Department of Neurology National Hospital Organization Hiroshima‐Nishi Medical Center Hiroshima Japan; ^7^ Memory Clinical Toride Toride Japan; ^8^ Biometrics Department Chugai Pharmaceutical Co., Ltd Tokyo Japan; ^9^ Pharmaceutical Science Department Chugai Pharmaceutical Co., Ltd Tokyo Japan; ^10^ Specialty Clinical Development Department Chugai Pharmaceutical Co., Ltd Tokyo Japan; ^11^ Department of Neurology Faculty of Medicine University of Tsukuba Tsukuba Japan

**Keywords:** Alzheimer's disease, amyloid positron emission tomography, anti‐amyloid beta, early Alzheimer's disease, gantenerumab, GRADUATE I, GRADUATE II, Japanese Alzheimer's disease participants, JP40959, mild Alzheimer's disease, mild cognitive impairment due to Alzheimer's disease, mild cognitive impairment, mild dementia, prodromal Alzheimer's disease

## Abstract

**INTRODUCTION:**

Gantenerumab was investigated in Japanese participants with mild cognitive impairment due to Alzheimer's disease (AD) or mild AD in two global phase 3 trials (GRADUATE I/II), and a phase 2 trial in Japan (JP40959).

**METHODS:**

Of 1965 participants randomized in GRADUATE I/II (global‐GRADUATE), 132 participants were enrolled from Japan (Japanese‐GRADUATE) and 67 Japanese participants were randomized 2:1:1 to high‐, low‐dose gantenerumab, and placebo in JP40959.

**RESULTS:**

Slowing of cognitive and functional decline, and amyloid reduction in gantenerumab group compared to placebo group were greater in Japanese‐GRADUATE than in the global‐GRADUATE and JP40959. Plasma gantenerumab concentrations in the Japanese‐GRADUATE were slightly higher than in the global‐GRADUATE and comparable to JP40959. Gantenerumab was well tolerated in the Japanese‐GRADUATE and JP40959, matching the safety profile of the global‐GRADUATE.

**DISCUSSION:**

Differences in results across the populations studied could be related to imbalances in baseline body weight, amyloid load, and disease severity.

**TRIAL REGISTRATION NUMBER:**

ClinicalTrials.gov ID: NCT03444870, NCT03443973; Japan Registry for Clinical Trials ID: jRCT2080224569.

**Highlights:**

- Gantenerumab was evaluated in Japanese participants with Alzheimer's disease (AD) in two global phase 3 trials and a phase 2 trial in Japan.
- Relative reduction in Clinical Dementia Rating Sum of Boxes (CDR‐SB) deterioration favored gantenerumab in Japanese‐GRADUATE (42%) more than in global‐GRADUATE (9%) and JP40959 (–24%).
- Amyloid reduction in Japanese‐GRADUATE was greater than in global‐GRADUATE and JP40959.
- Overall, 72.7% and 27.5% of Japanese‐ and global‐GRADUATE, respectively, achieved an amyloid‐negative status.
- Cognitive and functional decline, and amyloid reduction could be related to baseline body weight and disease severity.

## 1 BACKGROUND

Alzheimer's disease (AD) is a progressive, neurodegenerative disease associated with cognitive and functional decline.^1^ AD is the most common form of dementia, accounting for approximately two thirds of dementia cases, and the leading cause of long‐term care in Japan.^2^ In Japan, > 4.6 million people live with dementia and the lifetime risk of dementia in the Japanese elderly population is > 50%.^3^, ^4^ With the world's fastest aging population, the number of people in Japan living with dementia is expected to increase to 7 million by 2025.^4^, ^5^

The pathophysiology of AD is characterized by an extracellular accumulation of amyloid plaques, primarily composed of aggregated amyloid beta (Aβ) and intracellular neurofibrillary tangles, formed by aggregated pathologic tau protein, in the brain.^6^, ^7^ Evidence suggests that elevated Aβ levels promote the accumulation of neurotoxic forms of tau, eventually causing downstream neurodegeneration and subsequent dementia.^8^

Anti‐Aβ monoclonal antibodies have been developed and investigated in phase 2 and 3 clinical trials, with mixed results.^9^, ^10^, ^11^, ^12^, ^13^, ^14^, ^15^, ^16^ In trials that have reported the slowing of cognitive and functional decline, treatment with anti‐Aβ was associated with a high proportion of participants reaching amyloid negativity, as assessed by amyloid positron emission tomography (PET).^9^, ^10^, ^11^, ^13^, ^17^, ^18^ Antibodies which do not fully remove amyloid plaques have shown little to no benefit.^12^, ^14^, ^15^ To date, three molecules have received full approval for the treatment of AD based on evidence of amyloid‐lowering activity. Aducanumab was the first anti‐amyloid antibody to receive an accelerated approval by the US Food and Drug Administration (FDA) in June 2021 (although it was subsequently discontinued), followed by lecanemab in January 2023.^19^, ^20^, ^21^ The accelerated approval of lecanemab was converted into a traditional approval in July 2023^20^ and most recently, donanemab received a traditional FDA approval in July 2024.^22^

Gantenerumab is an investigational, fully human anti‐Aβ immunoglobulin G1 monoclonal antibody for subcutaneous administration, with a high affinity for aggregated Aβ, including oligomers, fibrils, and plaques. It removes Aβ via microglia‐mediated phagocytosis and has shown downstream effects on biomarkers of AD pathology and neurodegeneration in clinical trials.^23^, ^24^, ^25^ The efficacy and safety of gantenerumab was investigated in two global, phase 3, randomized trials, GRADUATE I (NCT03444870) and GRADUATE II (NCT03443973), for the treatment of early symptomatic AD.^16^ These studies did not meet their primary clinical outcome of slowing cognitive and functional decline as assessed by the change from baseline in the Clinical Dementia Rating Sum of Boxes (CDR‐SB) score between the gantenerumab and placebo groups at week 116.^16^ However, post hoc analyses of the findings of these studies showed East Asian participants, who had a lower baseline body weight and greater treatment compliance, showed a more favorable amyloid load reduction and slowing of cognitive and functional decline, compared to non‐East Asian participants.^26^ Gantenerumab was also investigated in a local phase 2 trial in Japan (JP40959; jRCT2080224569) designed to evaluate the dose–response effect of gantenerumab on amyloid load as measured by PET in Japanese participants with early symptomatic AD.

This article mainly focuses on data from the Japanese participants in the GRADUATE studies, alongside the global, overall population of the GRADUATE studies, and the Q2W double‐blind part of JP40959 (Figure S1 in supporting information), to evaluate whether the results for the East Asian subgroup can be extrapolated to Japanese people with early symptomatic AD.

## 2 METHODS

### 2.1 Trial design

**FIGURE 1 alz70192-fig-0001:**
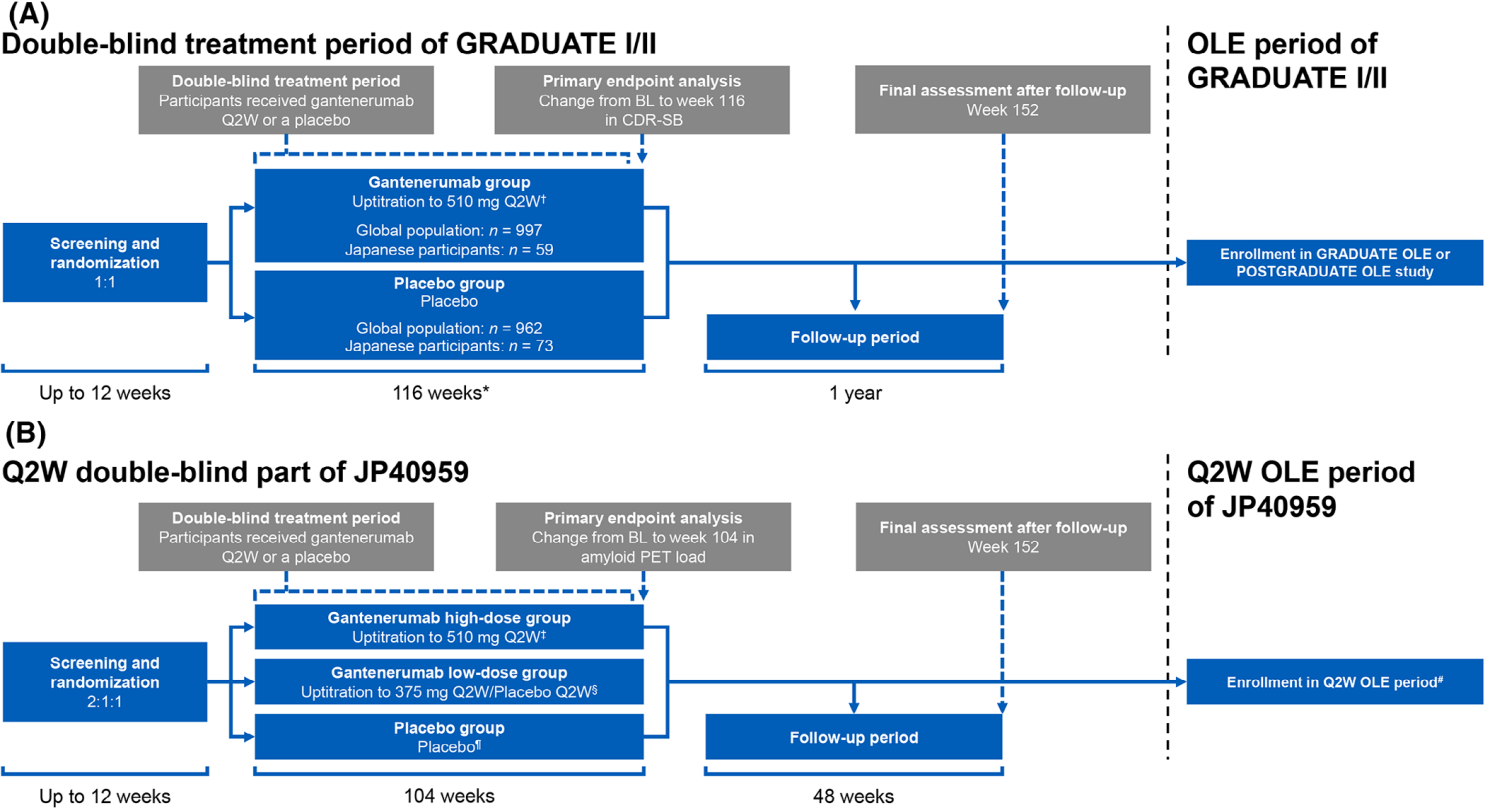
Study design of the GRADUATE studies (A) and JP40959 (B). *Extended to 116 weeks in response to delayed and missed visits due to the COVID‐19 pandemic. ^†^Participants randomized to the gantenerumab group received three doses of 120 mg Q4W, three doses of 255 mg Q4W, three doses of 510 mg Q4W, and 510 mg Q2W from week 36 onward. ^‡^Participants randomized to the high‐dose gantenerumab group received three doses of gantenerumab 120 mg Q4W, three doses of 255 mg Q4W, three doses of 510 Q4W, and 510 Q2W from week 36 onward. ^§^Participants randomized to the low‐dose gantenerumab group received three doses of gantenerumab 120 mg Q4W, three doses of 255 mg Q4W, three doses of 375 mg Q4W, and alternating doses of gantenerumab 375 mg and placebo Q2W from week 36 onward to maintain blinding the gantenerumab low‐dose group participants. ^¶^Participants randomized to the placebo group with identical dosing (excluding the active drug) to the high‐dose gantenerumab group. ^#^Participants were eligible for the Q2W OLE or Q1W OLE periods if they provided consent to participate in the respective OLE period, had not discontinued study treatment during the initial Q2W double‐blind or Q1W open‐label parts, and after the resolution of any ARIA MRI findings detected at week 104. ARIA, amyloid‐related imaging abnormalities; BL, baseline; CDR‐SB, Clinical Dementia Rating – Sum of Boxes; MRI, magnetic resonance imaging; OLE, open‐label extension; PET, positron emission tomography; Q1W, every week; Q2W, every 2 weeks; Q4W, every 4 weeks.

The GRADUATE studies were two phase 3, multicenter, randomized, double‐blind, placebo‐controlled, parallel‐group trials evaluating the efficacy and safety of gantenerumab in participants with early symptomatic AD^16^ (Figure 1A). Here, alongside the previously reported results in the global, overall population of the GRADUATE studies, we report the results of the Japanese participants for the double‐blind treatment period across 34 clinical sites (17 sites in GRADUATE I; 17 sites in GRADUATE II) and 7 amyloid PET imaging sites (4 sites in GRADUATE I; 3 sites in GRADUATE II). The double‐blind treatment period was initially planned to be 104 weeks and was extended to 116 weeks in response to delayed and missed visits due to the COVID‐19 pandemic.

JP40959 was a phase 2, multicenter, randomized, double‐blind, placebo‐controlled, parallel‐group trial evaluating the dose–response effect of gantenerumab on amyloid load as measured by PET in participants with early symptomatic AD. The trial was conducted across 18 clinical sites and 6 amyloid PET imaging sites in Japan. JP40959 consisted of two parts; a double‐blind part, in which participants received gantenerumab doses every 2 weeks (Q2W) or a placebo (Figure 1B), and an open‐label part, in which participants received gantenerumab doses every week (Q1W; Figure S2 in supporting information). The design for the Q2W double‐blind part except for having two dose groups of gantenerumab was similar to the GRADUATE studies, and enrollment began ≈ 1 year after the initiation of the GRADUATE studies. After up to 12 weeks of screening, eligible participants entered the treatment period for 104 weeks before the assessment of the primary endpoint of change from baseline in amyloid load as measured by PET at week 104. All participants who completed or discontinued any part entered an open‐label extension (OLE) period or a 48‐week follow‐up period. The details of the Q1W open‐label part and OLE period of JP40959 are provided in the Supplementary Methods in supporting information.

The protocol‐specified treatment durations at the target dose were 78 and 66 weeks in the GRADUATE studies and the Q2W double‐blind part of JP40959, respectively. The amyloid‐related imaging abnormality (ARIA) management algorithm for the Q2W double‐blind part of JP40959 was similar to that in the GRADUATE studies.^16^ ARIA magnetic resonance imaging (MRI) findings were reported as adverse events (AEs) if they met any of the following criteria: ARIA with edema (ARIA‐E) that was symptomatic, led to a change in study treatment, or was considered otherwise clinically significant according to the investigator's judgment.

**RESEARCH IN CONTEXT**

1. **Systematic review**: The authors reviewed literature obtained using search engines for biomedical literature (e.g., PubMed) and reviewed previous congress presentations of gantenerumab.
2. **Interpretation**: Compared to each placebo group, the slowing of cognitive and functional decline, and amyloid reduction were greater in Japanese participants in GRADUATE I and II (the GRADUATE studies) than in the global population of the GRADUATE studies and JP40959. These differences in the results could be related to imbalances in baseline body weight, amyloid load, and disease severity across the populations studied.
3. **Future directions**: Given the limited sample sizes of the trials and inconsistent results, further investigation of relationship between baseline body weight, amyloid load, and disease severity on cognition and function, and amyloid load reduction among Japanese patients with mild cognitive impairment due to Alzheimer's disease (AD) or mild AD is required.

As the GRADUATE studies did not meet their primary endpoints, JP40959 was terminated prior to the prespecified primary endpoint analysis. All participants in JP40959 discontinued dosing after it was decided on November 15, 2022 that the study would be terminated, and transitioned into a 12‐week, safety follow‐up period. The last participant's last visit was on February 27, 2023.

### 2.2 Trial oversight

The GRADUATE studies and JP40959 were sponsored by F. Hoffmann‐La Roche Ltd and Chugai Pharmaceutical Co., Ltd, respectively. The sponsor provided the trial drug and placebo. For the Japanese participants in the GRADUATE studies and JP40959, all authors made substantial contributions to design, acquisition, analysis, or interpretation of data for the work. All authors contributed to the development of the drafts with professional medical writing assistance, which was funded by Chugai Pharmaceutical Co., Ltd. The authors vouch for the completeness and accuracy of the data and for the fidelity of the trials to the protocol. Confidentiality agreements were in place between the authors and the sponsor of the respective studies, and the sponsor could not interdict or delay the publication of results. The trials were conducted in accordance with the International Council for Harmonisation E6 Guideline for Good Clinical Practice and the principles of the Declaration of Helsinki, as well as the laws and regulations of Japan, where the research was conducted. The protocol and any subsequent amendments were approved by the relevant institutional review board or ethics committee and by regulatory authorities. All trial participants provided written informed consent prior to study enrollment. An independent data and safety monitoring committee, which consisted of experts in AD and statistics, reviewed unblinded safety data during the GRADUATE studies and JP40959.[Fig alz70192-fig-0001]

### 2.3 Eligibility criteria

Eligibility criteria other than the amyloid plaque presence criteria for the double‐blind part of JP40959 were the same as for the GRADUATE studies. Eligible participants were between 50 and 90 years of age with a clinical diagnosis of probable AD according to the National Institute on Aging/Alzheimer's Association diagnostic criteria for mild AD, or prodromal AD (mild cognitive impairment [MCI] due to AD).^27^, ^28^ Participants needed to have a Mini‐Mental State Examination (MMSE) score of ≥ 22 (range 0–30, with lower scores indicating greater impairment); CDR – Global Score (GS) of 0.5 or 1.0 (possible scores are 0, 0.5, 1, 2, or 3, with higher scores indicating greater cognitive impairment); Free and Cued Selective Reminding Test (FCSRT) cueing index of ≤ 0.67 (range 0–1, with higher values indicating better performance) and an FCSRT free recall score of ≤ 27 (range 0–48, with higher values indicating better performance).^29^, ^30^ Participants must have evidence of amyloid pathology confirmed by a PET visual read or cerebrospinal fluid analysis (ratio of phosphorylated tau 181 to Aβ42 of > 0.024) in the GRADUATE studies, and/or PET visual read in JP40959.

Individuals were excluded if they had evidence of a condition other than AD that had the potential to affect cognitive function, as well as history or presence of psychiatric disorders, vascular disease, cerebrovascular disease, and systemic autoimmune disorders. Participants were also excluded based on imaging‐related criteria including clinically significant findings on MRI at screening, such as more than five microhemorrhages, more than two lacunar infarcts, or a Fazekas score of 3, indicating that confluent areas of the brain are affected by white matter hyperintensity; as well as the presence of any other significant cerebral abnormalities, including ARIA‐E, on screening MRI as assessed by the central reader.

### 2.4 Randomization and treatment administration

For the double‐blind period of the GRADUATE studies, eligible participants were randomized 1:1 to receive gantenerumab or placebo. The gantenerumab dose was up titrated over 36 weeks to a target level. Participants randomized to the gantenerumab group received a minimum of three doses at each level: three doses of 120 mg every 4 weeks (Q4W), three doses of 255 mg Q4W, and three doses of 510 mg Q4W, followed by the target dose of 510 mg Q2W from week 36 onward up to 116 weeks.^16^

For the Q2W double‐blind part of JP40959, eligible participants were 2:1:1 randomized to subcutaneously receive high‐dose gantenerumab, low‐dose gantenerumab, or a placebo, using a stratified block‐randomization method. Randomization to treatment group was stratified by stage of disease (MCI due to AD vs. mild AD dementia) and apolipoprotein E (*APOE*) ε4 genotype (carrier vs. non‐carrier) to maintain balance of these covariates between treatment groups. Similar to participants of the GRADUATE studies, participants in JP40959 randomized to gantenerumab groups had their gantenerumab doses up titrated over 36 weeks to a target level, receiving a minimum of three doses at each level. Participants randomized to the high‐dose gantenerumab group received at least three doses of gantenerumab 120 mg Q4W, three doses of 255 mg Q4W, and three doses of 510 mg Q4W, followed by the target dose of 510 mg Q2W from week 36 onward up to 104 weeks, which was the same regimen as the gantenerumab group in the GRADUATE studies. Participants randomized to the low‐dose gantenerumab group received three doses of gantenerumab 120 mg Q4W, three doses of 255 mg Q4W, and three doses of 375 mg Q4W, followed by alternating doses of gantenerumab 375 mg and placebo Q2W from week 36 onward, up to 104 weeks, to maintain blinding. Participants randomized to the placebo group received identical dosing (excluding the active drug) to the gantenerumab groups. Placebo group participants received nine doses of placebo Q4W, followed by placebo Q2W from week 36 onward, up to 104 weeks, to maintain blinding, which was the same regimen as that of the placebo group in the GRADUATE studies.

### 2.5 Endpoints

For the GRADUATE studies, the primary efficacy endpoint was the change from baseline to week 116 in CDR‐SB (range 0–18, with higher scores indicating greater cognitive impairment). Among others, secondary efficacy endpoints included the change from baseline to week 116 in the 13‐item cognitive subscale of the Alzheimer's Disease Assessment Scale (ADAS‐Cog13; range 0–85, with higher scores indicating greater cognitive impairment), Alzheimer's Disease Cooperative Study – Activities of Daily Living (ADCS‐ADL) total score (range 0–78, with lower scores indicating greater functional impairment), and MMSE. In the GRADUATE amyloid PET substudy, the change from baseline to week 116 in amyloid load and the proportion of participants who achieved amyloid‐negative status (defined as ≤ 24 Centiloid units [CL]) was assessed. The CL value of 24 is consistent with the diagnostic amyloid threshold that best distinguishes pathologically verified absence or sparse plaques from moderate to frequent plaques.^31^, ^32^

For JP40959, the primary endpoint was the change from baseline to week 104 in amyloid load as measured by PET standardized uptake value ratio; however, to compare the amyloid load as measured by PET between JP40959 and the GRADUATE studies, the results were converted to CL units. The change in cognition and function from baseline to week 104 as assessed by CDR‐SB, ADAS‐Cog13, ADCS‐ADL total score, and MMSE were evaluated as secondary and exploratory endpoints in the Q2W double‐blind part and the Q1W open‐label part, respectively.

Safety endpoints for the GRADUATE studies and JP40959 included the incidence, nature, severity, and timing of AEs, serious AEs (SAEs), injection‐site reactions, and ARIA MRI findings, including ARIA‐E and ARIA with hemosiderosis (ARIA‐H). The radiologic severity of ARIA was assessed with the Barkhof Grand Total Scale (BGTS; range 0–60, with higher scores indicating a greater extent of ARIA‐E).^33^ Pharmacokinetic (PK) endpoints for the GRADUATE studies and JP40959 were evaluated as a measure of gantenerumab plasma concentration at specified time points.

### 2.6 Statistical analysis

Analyses of the Japanese and global participants in the GRADUATE studies were based on pooled participant data across GRADUATE I and GRADUATE II. Subgroup analyses of the Japanese participants in the GRADUATE studies were performed using data for the primary analyses for the GRADUATE studies. For JP40959, the analyses including all data up to the last participant's last visit were conducted prior to a timing of prespecified primary analysis due to the study termination.

The number of Japanese participants to enroll in the GRADUATE studies and JP40959 was determined based on the notification published from Japanese Health Authority^34^ and the feasibility of conducting the study in Japan, respectively. Both were not based on statistical hypothesis testing. It was planned for 124 participants (gantenerumab, *n* = 62; placebo, *n* = 62) to be enrolled in the GRADUATE studies. For JP40959, 68 participants (high‐dose gantenerumab, *n *= 34; low‐dose gantenerumab, *n *= 17; placebo, *n *= 17) were planned to be enrolled in the Q2W double‐blind part, and subsequently, 12 participants to be enrolled in the Q1W open‐label part.

For the Japanese participants in the GRADUATE studies and the Q2W double‐blind part of JP40959, efficacy analyses were performed on all randomized participants who received at least one dose of the study drug, grouped according to the assigned treatment group. The endpoints for cognition and function, and amyloid load were assessed using mixed models for repeated measures (MMRM) with adjustment for the prespecified covariates including Study ID (GRADUATE I vs. GRADUATE II; only for the GRADUATE studies), stage of disease (MCI due to AD vs. mild AD dementia), *APOE* ε4 carrier status (carrier or non‐carrier), AD medication at baseline, the baseline score, visit, treatment group, or treatment group by visit interaction as fixed effects. The MMRM analysis model was used in both GRADUATE studies and JP40959 as a sensitivity analysis and a primary analysis, respectively. Safety analyses were performed on all randomized participants who received at least one dose of the study drug, grouped according to treatment received. Safety data were summarized by treatment received. PK analyses were performed on all participants randomized to the gantenerumab group in the GRADUATE studies, and the high‐dose or low‐dose gantenerumab groups in JP40959, who received at least one dose of the study drug. Summary statistics and time courses were presented for gantenerumab plasma concentrations. Of note, all gantenerumab plasma concentrations measured after at least one irregular dose (modification, interruption, or discontinuation) were excluded from PK data summarization.

## 3 RESULTS

### 3.1 Trial participant population and drug exposure

For the Japanese participants in the GRADUATE studies, 132 participants (gantenerumab, *n *= 59; placebo, *n *= 73) were randomized and treated. Of these, 119 participants (gantenerumab, *n *= 53 [89.8%]; placebo, *n *= 66 [90.4%]) completed the double‐blind treatment period (Figure S3A in supporting information). The proportion of Japanese participants who completed the double‐blind treatment period was slightly higher than that of the global population of the GRADUATE studies (119 participants [90.2%] vs. 1531 participants [77.9%]).^16^ For the Q2W double‐blind part of JP40959, 67 participants were randomized and treated (high‐dose gantenerumab, *n *= 34; low‐dose gantenerumab, *n *= 17; placebo, *n *= 16), and 58 completed the study or discontinued due to the study's termination by sponsor (high‐dose gantenerumab, *n *= 31 [91.2%]; low‐dose gantenerumab, *n *= 14 [82.4%]; placebo, *n *= 13 [81.3%]; Figure S3B).

Baseline demographics for the Japanese participants and the global population of the GRADUATE studies were comparable between studies and treatment groups except for body weight (Table 1). Japanese participants had a lower mean body weight in the GRADUATE studies (gantenerumab: 52.74 kg; placebo: 52.96 kg) than the global population of the GRADUATE studies (gantenerumab: 69.38 kg; placebo, 68.18 kg). For the Q2W double‐blind part of JP40959, body weight (high‐dose gantenerumab: 54.99 kg; low‐dose gantenerumab: 52.51 kg; placebo: 50.47 kg) was comparable to the Japanese participants in the GRADUATE studies. In addition, the distribution of the *APOE* ε4 carriers (1 ε4 and 2 ε4) and non‐carriers in groups except for the placebo group (high‐dose gantenerumab: *n* = 7 [20.6%]; low‐dose gantenerumab: *n* = 2 [11.8%]) were comparable to the Japanese participants and the global population of the GRADUATE studies, whereas there were no homozygous *APOE* ε4 carriers in the placebo group.

**TABLE 1:** Baseline demographics and disease characteristics for participants in GRADUATE studies and the Q2W double‐blind part of JP40959.

	GRADUATE studies				JP40959		
	Japanese participants		Global population		Q2W double‐blind part		
Characteristic	Gantenerumab (*n *= 59)	Placebo (*n* = 73)	Gantenerumab (*n* = 997)	Placebo (*n* = 962)	High‐dose gantenerumab (*n* = 34)	Low‐dose gantenerumab (*n* = 17)	Placebo (*n* = 16)
Age, years, mean (SD)	73.3 (7.0)	73.0 (7.5)	71.4 (7.9)	72.0 (7.6)	71.3 (7.7)	74.1 (5.3)	72.3 (8.0)
Sex, Female, *n* (%)	34 (57.6)	42 (57.5)	578 (58.0)	540 (56.1)	19 (55.9)	12 (70.6)	12 (75.0)
Race, Asian, *n* (%)	59 (100.0)	73 (100.0)	108 (10.8)	128 (13.3)	34 (100.0)	17 (100.0)	16 (100.0)
Body weight, kg, mean (SD)	52.74 (8.16)	52.96 (8.34)	69.38 (14.68)	68.18 (13.74)	54.99 (7.70)	52.51 (9.86)	50.47 (10.72)
BMI, kg/m^2^, mean (SD)	21.48 (2.96)	21.35 (2.52)	25.50 (4.27)	25.18 (4.48)	21.77 (2.35)	21.37 (2.67)	20.67 (3.46)
*APOE* ε4 allele, *n* (%)							
0 ɛ4	21 (35.6)	25 (34.2)	338 (33.9)	313 (32.5)	12 (35.3)	6 (35.3)	5 (31.3)
1 ɛ4	27 (45.8)	41 (56.2)	477 (47.8)	495 (51.5)	15 (44.1)	9 (52.9)	11 (68.8)
2 ɛ4	11 (18.6)	7 (9.6)	182 (18.3)	154 (16.0)	7 (20.6)	2 (11.8)	0 (0.0)
Use of AD medication at enrollment, *n* (%)	33 (55.9)	53 (72.6)	643 (64.5)	610 (63.4)	22 (64.7)	8 (47.1)	10 (62.5)
AD diagnosis at screening, *n* (%)							
MCI due to AD	42 (71.2)	45 (61.6)	544 (54.6)	529 (55.0)	15 (44.1)	8 (47.1)	7 (43.8)
Mild AD dementia	17 (28.8)	28 (38.4)	453 (45.4)	433 (45.0)	19 (55.9)	9 (52.9)	9 (56.3)
CDR‐GS, *n* (%)							
0.5	49 (83.1)	56 (76.7)	688 (69.1)	719 (74.7)	28 (82.4)	14 (82.4)	15 (93.8)
1	10 (16.9)	17 (23.3)	299 (30.0)	239 (24.8)	6 (17.6)	3 (17.6)	1 (6.3)
MMSE, mean (SD)	24.1 (2.9)	24.0 (2.5)	23.6 (3.2)	23.7 (3.1)	23.2 (3.0)	23.4 (3.5)	23.8 (2.7)
FCSRT, mean (SD)							
Free recall	7.7 (5.4)	7.9 (5.7)	9.0 (5.6)	8.6 (5.5)	7.5 (4.4)	8.1 (6.4)	7.4 (5.1)
Cueing index	0.419 (0.168)	0.381 (0.145)	0.434 (0.143)	0.426 (0.148)	0.402 (0.181)	0.353 (0.158)	0.345 (0.148)
CDR‐SB, mean (SD)	3.08 (1.25)	3.34 (1.24)	3.69 (1.64)	3.61 (1.56)	3.43 (1.05)	3.47 (1.66)	2.78 (1.09)
ADAS‐Cog13, mean (SD)	27.3 (5.4)	27.7 (6.1)	28.1 (7.0)	28.1 (6.9)	27.2 (7.0)	27.7 (5.0)	26.7 (3.8)
ADCS‐ADL total score, mean (SD)	67.7 (5.6)	68.3 (5.9)	68.1 (7.3)	68.6 (7.0)	65.1 (6.9)	68.0 (6.4)	68.0 (7.1)
Baseline amyloid load as measured by PET, mean CL (SD)	81.04 (22.39)^a^	79.56 (24.83)^a^	94.70 (27.04)^b^	94.23 (31.87)^b^	84.99 (23.40)	85.23 (16.88)	86.54 (22.12)

*Note*: Assessment scoring ranges: CDR‐GS, 0–3; MMSE, 0–30; FCSRT free recall, 0–48; FCSRT Cueing index, 0.0–1.0; CDR‐SB,0–18; ADAS‐Cog13, 0–85; ADCS‐ADL total score, 0–78.

Abbreviations: AD, Alzheimer's disease; ADAS‐Cog13, 13‐item cognitive subscale of the Alzheimer's Disease Assessment Scale; ADCS‐ADL, Alzheimer's Disease Cooperative Study – Activities of Daily Living; *APOE* *ɛ*4, apolipoprotein E *ɛ*4 allele; BMI, body mass index; CDR‐GS, Clinical Dementia Rating – Global Score; CDR‐SB, Clinical Dementia Rating – Sum of Boxes; CL, Centiloid; FCSRT, Free and Cued Selective Reminding Test; MCI, mild cognitive impairment; MMSE, Mini‐Mental State Examination; PET, positron emission tomography; Q2W, every 2 weeks; SD, standard deviation.

^a^ A total of 30 Japanese participants in the GRADUATE studies (gantenerumab group, *n* = 14; placebo group, *n* = 16) were enrolled in the amyloid PET substudy.

^b^ A total of 308 participants in the global population of the GRADUATE studies (gantenerumab group, *n* = 160; placebo group, *n* = 148) were enrolled in the amyloid PET substudy.

Disease characteristics differed between treatment groups for the Japanese participants and the global population of the GRADUATE studies (Table 1). Specifically, the proportion of AD medication usage at baseline was lower in the gantenerumab group (*n* = 33 [55.9%]) of Japanese participants in the GRADUATE studies versus the placebo group (*n* = 53 [72.6%]), and both groups in the global population of the GRADUATE studies (gantenerumab: *n* = 643 [64.5%]; placebo: *n* = 610 [63.4%]). In addition to an imbalance between treatment groups, the proportion of MCI due to AD was higher in the Japanese participants in the GRADUATE studies (gantenerumab: *n* = 42 [71.2%]; placebo: *n* = 45 [61.6%]) than the global population of the GRADUATE studies (gantenerumab: *n* = 544 [54.6%]; placebo: *n* = 529 [55.0%]). Baseline mean (standard deviation [SD]) CDR‐SB scores were lower in the gantenerumab group of Japanese participants in the GRADUATE studies (3.08 [1.25]) versus the placebo group (3.34 [1.24]), and both groups in the global population of the GRADUATE studies (gantenerumab: 3.69 [1.64]; placebo: 3.61 [1.56]). Baseline mean (SD) amyloid PET load (CL) was numerically lower in Japanese participants in the GRADUATE studies (gantenerumab: 81.04 [22.39]; placebo: 79.56 [24.83]) versus the global population of the GRADUATE studies (gantenerumab: 94.70 [27.04]; placebo: 94.23 [31.87]). For the Q2W double‐blind part of JP40959, the proportion of MCI due to AD (high‐dose gantenerumab: *n* = 15 [44.1%]; low‐dose gantenerumab: 8 [47.1%]; placebo: *n* = 7 [43.8%]) was lower than the Japanese participants and the global population of the GRADUATE studies, whereas baseline mean (SD) amyloid PET load (CL; high‐dose gantenerumab: 84.99 [23.40]; low‐dose gantenerumab; 85.23 [16.88]; placebo: 86.54 [22.12]) was between the Japanese participants and the global population of the GRADUATE studies. In addition to disease stage imbalance between studies, baseline mean (SD) CDR‐SB in placebo group (2.78 [1.09]) was lower than other groups (high‐dose gantenerumab: 3.43 [1.05]; low‐dose gantenerumab; 3.47 [1.66]). The baseline demographics and characteristics of the Q1W open‐label participants in JP40959 are presented in Table S1 in supporting information.

The mean (SD) treatment durations at the target dose for the gantenerumab group of Japanese participants and the global population of the GRADUATE were 74.0 (12.5) and 65.3 (21.46) weeks, respectively, and that of the Q2W double‐blind part of JP40959 was 57.19 (15.36; Table S2 in supporting information). The differences between the protocol‐specified and actual treatment durations were larger in JP40959 than the Japanese participants in the GRADUATE studies due to JP40959 study termination by the sponsor. The results for the Q1W open‐label part and OLE period of JP40959 are presented in the Supplementary Results in supporting information.

### 3.2 Effect of gantenerumab on cognition and function

**FIGURE 2:**
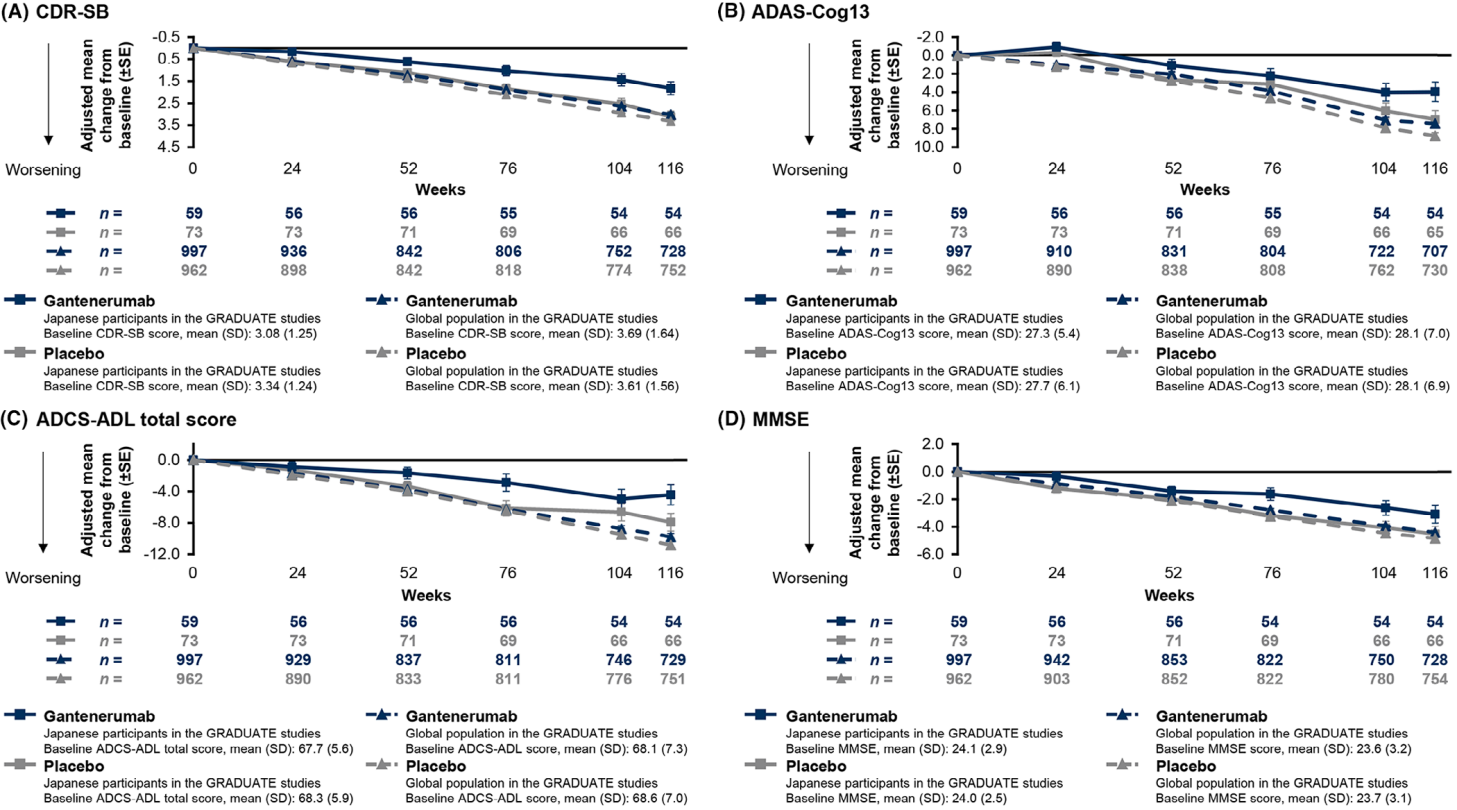
Adjusted mean change from baseline in cognitive and functional endpoints in the GRADUATE studies. These clinical endpoints were assessed using an MMRM model; the details of the model have been reported previously.^16^ For Japanese participants in the GRADUATE studies, the MMRM model excluded the geographic region as a fixed covariate that was used for the global population. ADAS‐Cog13, 13‐item cognitive subscale of the Alzheimer's Disease Assessment Scale; ADCS‐ADL, Alzheimer's Disease Cooperative Study – Activities of Daily Living; CDR‐SB, Clinical Dementia Rating – Sum of Boxes; MMRM, mixed models for repeated measures; MMSE, Mini‐Mental State Examination; SD, standard deviation; SE, standard error.

For the Japanese participants in the GRADUATE studies, the adjusted mean (standard error [SE]) change from baseline in CDR‐SB at week 116 was 1.83 (0.296) in the gantenerumab group and 3.13 (0.265) in the placebo group (difference from placebo group [95% confidence interval (CI)], –1.31 [−2.01 to −0.51]; relative reduction in worsening of CDR‐SB compared to placebo group, 42%), which showed a favorable difference relative to the global population of the GRADUATE studies (difference from placebo group [95% CI], −0.29 [−0.58 to −0.01]; relative reduction, 9%; Figure 2A, Table S3 in supporting information). In the Q2W double‐blind part of JP40959, the adjusted mean (SE) changes from baseline in CDR‐SB at week 104 were 2.05 (0.411) in the high‐dose gantenerumab group, 2.04 (0.576) in the low‐dose gantenerumab group, and 1.65 (0.617) in the placebo group (differences from placebo group [95% CI], 0.40 [−1.08 to 1.87] and 0.39 [−1.30, 2.07], respectively; relative reduction in worsening of CDR‐SB compared to placebo group, −24% and −23%, respectively). Point estimates showing changes in CDR‐SB from baseline to Week 104 favored the placebo group (Table S3, Figure S4A in supporting information). Trends in the results of ADAS‐Cog13 (Figure 2B, Figure S4B), ADCS‐ADL (Figure 2C, Figure S4C), and MMSE (Figure 2D, Figure S4D) for the GRADUATE studies and JP40959 were consistent with CDR‐SB.

### 3.3 Effect of gantenerumab on amyloid load as measured by PET

**FIGURE 3:**
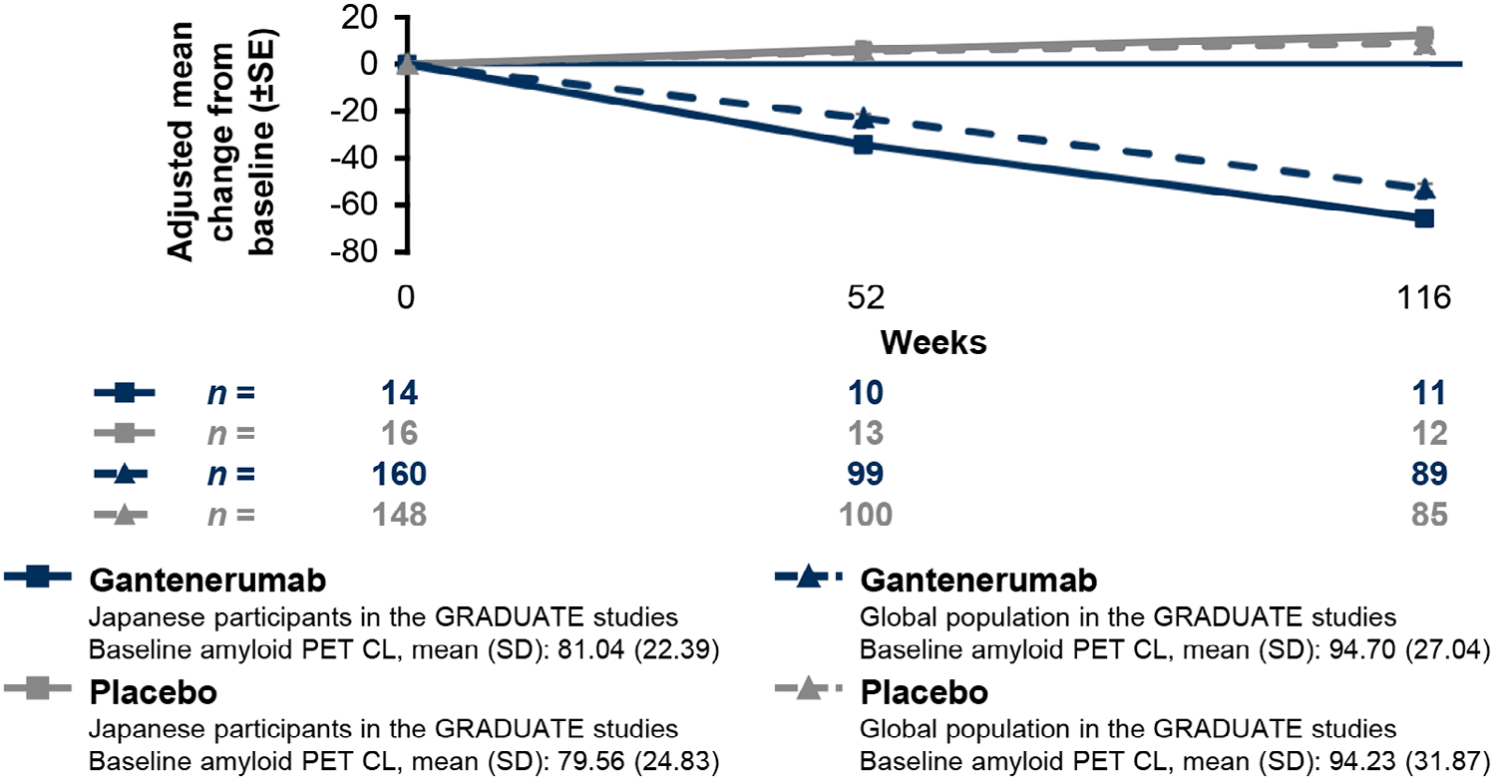
Adjusted mean change from baseline in amyloid load as measured by PET in the GRADUATE studies. The change from baseline in amyloid load was assessed using an MMRM model with fixed covariates of baseline amyloid load value, *APOE* ε4 carrier status (carrier or non‐carrier), visit, treatment group, and treatment group by visit interaction. For global population of the GRADUATE studies these clinical endpoints were assessed using an MMRM model; the details of the model have been reported previously.^16^ For Japanese participants in GRADUATE studies, the MMRM model excluded type of tracer as a covariate that was used for global population. *APOE* ε4, apolipoprotein E ε4 allele; CL, Centiloid; MMRM, mixed models for repeated measures; PET, positron emission tomography; SD, standard deviation; SE, standard error.

Of the Japanese participants and the global population of the GRADUATE studies, 30 participants (gantenerumab group, *n* = 14; placebo group, *n* = 16) and 308 participants (gantenerumab group, *n* = 160; placebo group, *n* = 148) were enrolled in the amyloid PET substudy. The amyloid load, as measured by PET, at week 116 for the gantenerumab group in the Japanese participants in the GRADUATE studies was lower than the amyloid load for that in the global population of the GRADUATE studies (Figure 3). As well, the adjusted mean (SE) change from baseline to week 116 in amyloid load (CL) for the gantenerumab group in the Japanese participants in the GRADUATE studies was −65.80 (3.593; placebo: 12.23 [3.401]; difference from placebo group [95% CI], −78.02 [−88.52 to −67.53]), which was greater than that in the global population of the GRADUATE studies (gantenerumab: −53.08 [2.001], placebo: 8.84 [2.039]; difference from placebo group [95% CI], −61.92 [−67.56 to −56.28]). In the Q2W double‐blind part of JP40959, the adjusted mean (SE) change from baseline to week 104 in amyloid load (CL) was −53.83 (3.494) in the high‐dose gantenerumab group, –39.40 (5.098) in the low‐dose gantenerumab group, and 6.35 (5.221) in the placebo group (differences from placebo group [95% CI], −60.18 [−72.59 to −47.76] and −45.75 [−60.17 to −31.33], respectively; Figure S5, Table S3 in supporting information). The reduction in amyloid load from baseline to week 104 for the Q2W double‐blind part of JP40959 was dose dependent. The mean values for amyloid load on PET in Japanese participants in the GRADUATE studies and participants of JP40959 are shown in Figure S6 in supporting information.

The proportion of participants who achieved amyloid‐negative status was 72.7% (8 of 11 participants) at week 116 in the gantenerumab group of Japanese participants in the GRADUATE studies, which was higher than that in the gantenerumab group of the global population of the GRADUATE studies (27.5%, 25 of 91 participants; Table 2). In the high‐ and low‐dose gantenerumab groups of JP40959, the proportions of participants with amyloid‐negative status at week 104 were 37.5% (9 of 24) and 8.3% (1 of 12), respectively (Table S4 in supporting information).

**TABLE 2:** Proportion of participants achieving amyloid positivity threshold by visit week in the GRADUATE studies.

	Japanese participants in the GRADUATE studies^a^		Global population of the GRADUATE studies^b^	
	Gantenerumab (*n* = 14)	Placebo (*n* = 16)	Gantenerumab (*n* = 160)	Placebo (*n* = 148)
**Baseline**				
*n*	14	16	160	148
Baseline amyloid load as measured by PET, mean CL (SD)	81.04 (22.39)	79.56 (24.83)	94.70 (27.04)	94.23 (31.87)
≤24 CL, *n* (%)	0 (0.0)	0 (0.0)	2 (1.3)	3 (2.0)
>24 CL, *n* (%)	14 (100)	16 (100)	158 (98.8)	145 (98.0)
**Week 52**				
*n*	10	13	100	100
≤24 CL, *n* (%)	0 (0.0)	0 (0.0)	2 (2.0)	1 (1.0)
>24 CL, *n* (%)	10 (100)	13 (100)	98 (98.0)	99 (99.0)
**Week 116**				
*n*	11	12	91	87
≤24 CL, *n* (%)	8 (72.7)	0 (0.0)	25 (27.5)	1 (1.1)
>24 CL, *n* (%)	3 (27.3)	12 (100)	66 (72.5)	86 (98.9)

*Note*: Amyloid positivity is defined as ≤ 24 CL.

Abbreviations: CL, Centiloid; PET, positron emission tomography; SD, standard deviation.

^a^ A total of 30 Japanese participants in the GRADUATE studies (gantenerumab group, *n* = 14; placebo group, *n* = 16) were enrolled in the amyloid PET substudy.

^b^ A total of 308 participants in the global population of the GRADUATE studies (gantenerumab group, *n* = 160; placebo group, *n* = 148) were enrolled in the amyloid PET substudy.

The effect of gantenerumab on amyloid load for the Q1W open‐label part and OLE period of JP40959 are presented in Supplementary Results.

### 3.4 Pharmacokinetics

**FIGURE 4 alz70192-fig-0004:**
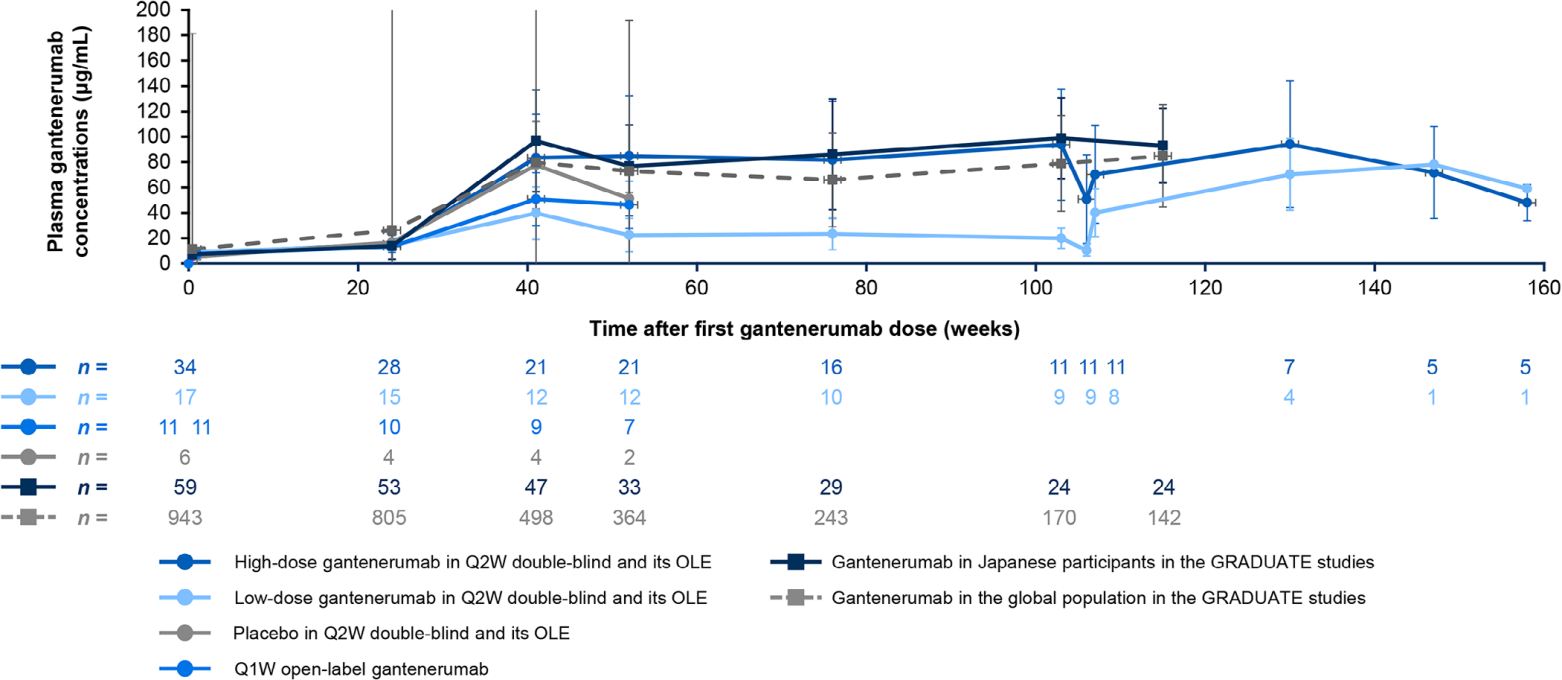
Mean ± SD gantenerumab plasma concentration–time course from first gantenerumab dose in the GRADUATE studies and JP40959, including OLE period. If more than half of the measured values were below the limit of quantification (i.e., 0.500 ng/mL) at a sampling time point, the mean and SD were not calculated. OLE, open‐label extension; Q1W, every week; Q2W, every 2 weeks; SD, standard deviation.

In the Q2W double‐blind part of JP40959, dose‐dependent increases in mean plasma gantenerumab concentrations were observed at and after week 41 in the high‐ and low‐dose gantenerumab groups. Mean plasma gantenerumab concentrations in the Q1W open‐label part were ≈ 40% lower than in the high‐dose gantenerumab group. Mean plasma gantenerumab concentrations at steady state in the high‐dose gantenerumab group and the Q2W OLE period of JP40959 were comparable. PK profiles were comparable between the high‐dose gantenerumab group of JP40959 and the Japanese participants in the GRADUATE studies (Figure 4, Table S5 in supporting information).

### 3.5 AEs and ARIA MRI findings

In this section, we focus on data from Japanese participants of GRADUATE studies and JP40959, alongside the global population of the GRADUATE studies. An overview of the AEs that occurred in the double‐blind period of GRADUATE studies and Q2W double‐blind part of JP40959 is provided in Table 3.[Fig alz70192-fig-0004], [Table alz70192-tbl-0003] An overview of AEs that occurred after initial gantenerumab administration in the Japanese participants in the GRADUATE studies and JP40959 including its OLE periods is provided in Table S6 in supporting information. The most common AEs with an incidence rate of ≥ 10% in any treatment group of the GRADUATE studies and the Q2W double‐blind and Q1W open‐label parts of JP40959, and after initial gantenerumab administration in JP40959 are reported in Tables S7 and S8 in supporting information, respectively. ARIA‐E, ARIA‐H, and injection‐site reactions are identified risks of gantenerumab.

**TABLE 3 alz70192-tbl-0003:** Overview of adverse events in GRADUATE studies and the Q2W double‐blind part of JP40959.

	Japanese participants in the GRADUATE studies		Q2W double‐blind part of JP40959			Global population of the GRADUATE studies	
	Gantenerumab (*n* = 59)	Placebo (*n* = 73)	High‐dose gantenerumab (*n* = 34)	Low‐dose gantenerumab (*n* = 17)	Placebo (*n* = 16)	Gantenerumab (*n* = 1004)	Placebo (*n* = 955)
Participants who experienced an AE, *n* (%)	54 (91.5)	69 (94.5)	31 (91.2)	16 (94.1)	14 (87.5)	905 (90.1)	832 (87.1)
Participants who experienced an SAE, *n* (%)	4 (6.8)	20 (27.4)	5 (14.7)	4 (23.5)	0 (0.0)	137 (13.6)	158 (16.5)
Participants permanently discontinuing treatment due to an AE, *n* (%)	6 (10.2)	1 (1.4)	0 (0.0)	1 (5.9)	1 (6.3)	91 (9.1)	17 (1.8)
Participants who experienced an AE with a fatal outcome, *n* (%)	0 (0.0)	1 (1.4)	0 (0.0)	1 (5.9)	0 (0.0)	10 (1.0)	14 (1.5)
Participants who experienced an AE related to study treatment, *n* (%)	22 (37.3)	8 (11.0)	14 (41.2)	7 (41.2)	4 (25.0)	417 (41.5)	152 (15.9)
ARIA‐E, *n* (%)^a^	12 (20.3)	3 (4.1)	6 (17.6)	3 (17.6)	1 (6.3)	219 (21.8)	17 (1.8)
ARIA‐H, *n* (%)^b^	4 (6.8)	1 (1.4)	1 (2.9)	4 (23.5)	1 (6.3)	78 (7.8)	9 (0.9)
ISR, *n* (%)	4 (6.8)	2 (2.7)	4 (11.8)	2 (11.8)	0 (0.0)	169 (16.8)	74 (7.7)

Abbreviations: AE, adverse event; ARIA‐E, amyloid‐related imaging abnormalities – edema; ARIA‐H, amyloid‐related imaging abnormalities – hemosiderosis; ISR, injection‐site reaction; Q2W, every 2 weeks; SAE, serious adverse event.

^a^ ARIA‐E includes the events “amyloid related imaging abnormality‐edema/effusion,” “vasogenic cerebral edema,” and “brain edema.” ARIA‐E had to be reported as an AE if it was symptomatic, led to dosing intervention, or was otherwise considered clinically significant by the investigator.

^b^ ARIA‐H includes the events “amyloid related imaging abnormality‐microhemorrhages and hemosiderin deposits,” “cerebral microhemorrhage,” “cerebral hemosiderin deposition,” “cerebellar microhemorrhage,” and “brain stem microhemorrhage.” ARIA‐H had to be reported as an AE if it led to dosing intervention or was otherwise considered clinically significant by the investigator.

ARIA MRI findings are reported independently of AEs (Table 4). The incidences of ARIA‐E MRI findings were 24.1% (14 of 58) in the gantenerumab group and 6.8% (5 of 73) in the placebo group in the Japanese participants in the GRADUATE studies, and 17.6% (6 of 34) in the high‐dose gantenerumab group, 17.6% (3 of 17) in the low‐dose gantenerumab group, and 6.3% (1 of 16) in the placebo group in the Q2W double‐blind part of JP40959. The incidences of ARIA‐E MRI findings were 24.9% (247 of 993) in the gantenerumab group and 2.7% (26 of 946) in the placebo group in the global population of the GRADUATE studies. Severity of ARIA‐E MRI findings occurring for the Japanese participants in the GRADUATE studies, the Q2W double‐blind part, and Q1W open‐label part of JP40959 were comparable to those of the global population of the GRADUATE studies (Table 4, Table S9 in supporting information). The incidence of ARIA‐H MRI findings concurrent with ARIA‐E MRI findings was greater between gantenerumab in Q2W double‐blind part of JP40959 (high‐dose gantenerumab: 8.8% [3 of 34]; low‐dose gantenerumab: 17.6% [3 of 17]) and in the Japanese participants in the GRADUATE studies (5.2% [3 of 58]) versus placebo in Q2W double‐blind part of JP40959 (0 of 16) and in the Japanese participants in the GRADUATE studies (2.7% [2 of 73]), respectively. However, the incidence of isolated ARIA‐H MRI findings had no clear differences between groups in the Japanese participants in the GRADUATE studies (gantenerumab: 13.8% [8 of 58]; placebo: 11.0% [8 of 73]) and the Q2W double‐blind part of JP40959 (high‐dose gantenerumab: 11.8% [4 of 34]; low‐dose gantenerumab: 11.8% [2 of 17]; placebo: 6.3% [1 of 16]; Table 4).

**TABLE 4:** Incidence of ARIA MRI findings in the GRADUATE studies and the Q2W double‐blind part of JP40959.

	Japanese participants in the GRADUATE studies		Q2W double‐blind part of JP40959			Global participants in the GRADUATE studies	
	Gantenerumab (*n* = 58)	Placebo (*n* = 73)	High‐dose gantenerumab (*n* = 34)	Low‐dose gantenerumab (*n* = 17)	Placebo (*n* = 16)	Gantenerumab (*n* = 993)	Placebo (*n* = 946)
Incidence of ARIA‐E, *n* (%)	14 (24.1)	5 (6.8)	6 (17.6)	3 (17.6)	1 (6.3)	247 (24.9)	26 (2.7)
ARIA‐E by *APOE* ε4 genotype, *n*/total *n* (%)							
0 ε4	3/21 (14.3)	3/25 (12.0)	2/12 (16.7)	1/6 (16.7)	0/5 (0.0)	44/335 (13.1)	9/310 (2.9)
1 ε4	6/26 (23.1)	2/41 (4.9)	3/15 (20.0)	1/9 (11.1)	1/11 (9.1)	117/478 (24.5)	10/486 (2.1)
2 ε4	5/11 (45.5)	0/7 (0.0)	1/7 (14.3)	1/2 (50.0)	0/0 (0.0)	86/180 (47.8)	7/150 (4.7)
Recurrent ARIA‐E, *n* (%)	5 (8.6)	0 (0.0)	2 (5.9)	2 (11.8)	0 (0.0)	95 (9.6)	3 (0.3)
Symptomatic ARIA‐E, *n* (%)^a^	0 (0.0)	0 (0.0)	0 (0.0)	2 (11.8)^b^	0 (0.0)	50 (5.0)	2 (0.2)
Serious symptomatic ARIA‐E, *n* (%)^c^	0 (0.0)	0 (0.0)	0 (0.0)	0 (0.0)	0 (0.0)	11 (1.1)	0 (0.0)
Most severe ARIA‐E (BGTS)							
Mean (SD)	8.6 (7.3)	2.2 (1.8)	11.0 (5.7)	15.7 (6.4)	1.0 (NE)	10.6 (8.4)	3.3 (2.9)
≥4, *n*/total *n* (%)^d^	11/14 (78.6)	1/5 (20.0)	5/6 (83.3)	3/3 (100.0)	0/1 (0.0)	203/247 (82.2)	8/26 (30.8)
Baseline ARIA‐H, *n* (%)							
0	52 (89.7)	65 (89.0)	28 (82.4)	13 (76.5)	15 (93.8)	896 (90.2)	834 (88.3)
1–5	6 (10.3)	8 (11.0)	6 (17.6)	4 (23.5)	1 (6.3)	96 (9.7)	111 (11.7)
>5	0 (0.0)	0 (0.0)	0 (0.0)	0 (0.0)	0 (0.0)	1 (0.1)	0 (0.0)
Overall incidence of ARIA‐H, *n* (%)^e^	11 (19.0)	10 (13.7)	8 (23.5)	5 (29.4)	1 (6.3)	227 (22.9)	116 (12.3)
Incidence of ARIA‐H concurrent with ARIA‐E, *n* (%)^f^	3 (5.2)	2 (2.7)	3 (8.8)	3 (17.6)	0 (0.0)	134 (13.5)	7 (0.7)
Incidence of isolated ARIA‐H, *n* (%)^g^	8 (13.8)	8 (11.0)	4 (11.8)	2 (11.8)	1 (6.3)	85 (8.6)	108 (11.4)

*Note*: Data for participants who underwent MRI after baseline are shown; those who discontinued the trial before assessment are not included.

Abbreviations: AE, adverse event; *APOE* *ɛ*4, apolipoprotein E *ɛ*4 allele; ARIA, amyloid‐related imaging abnormalities; ARIA‐E, amyloid‐related imaging abnormalities – edema; ARIA‐H, amyloid‐related imaging abnormalities – hemosiderosis; BGTS, Barkhof Grand Total Scale; CNS, central nervous system; MRI, magnetic resonance imaging; NE, not evaluable; Q2W, every 2 weeks; SD, standard deviation.

^a^ Symptomatic ARIA‐E is defined as ARIA‐E temporally associated with CNS symptoms. All CNS symptoms associated with ARIA‐E were resolved.

^b^ Total of three symptomatic ARIA‐E in two participants were reported. Symptomatic ARIA‐E were as follows: aphasia, headache, dizziness.

^c^ Either the ARIA‐E or CNS symptom(s) were reported as a serious AE.

^d^ BGTS score ≥ 4 is considered radiologically moderate or severe.

^e^ The counts represent cumulative post‐baseline findings, excluding any corresponding ARIA‐H at baseline.

^f^ Concurrence is defined as temporal co‐occurrence, with new ARIA‐H MRI findings detected at the same time as emerging or ongoing ARIA‐E MRI findings.

^g^ Participants who did not develop incident ARIA‐E during the study period.

The incidence of injection‐site reactions was, for the Japanese participants in the GRADUATE studies, 6.8% (4 of 59) in gantenerumab group and 2.7% (2 of 73) in placebo group, and for the Q2W double‐blind part of JP40959, 11.8% (4 of 34) in the high‐dose gantenerumab group, 11.8% (2 of 17) in the low‐dose gantenerumab group, and 0% in the placebo group (Table 3).

Incidences of AEs that occurred in > 10% of the Japanese participants in the GRADUATE studies, the Q2W double‐blind part, and the Q1W open‐label part of JP40959 were comparable to those of the overall of the GRADUATE studies (Table S7 in supporting information). No new safety signals were detected in the entire period of JP40959 (Tables S6–S8 in supporting information).

## 4 DISCUSSION

This report is the first to evaluate the efficacy, safety, and PK outcomes of gantenerumab in Japanese participants with early AD, who enrolled in the GRADUATE studies and JP40959. Favorable effects in slowing cognitive and functional decline, as assessed by CDR‐SB, ADAS‐Cog13, ADCS‐ADL, and MMSE, and amyloid load reduction in gantenerumab‐treated Japanese participants in the GRADUATE studies were observed (Figures 2 and 3, Table S3).

The Japanese population in the GRADUATE studies who experienced favorable effects in slowing cognitive and functional decline, and a greater amyloid reduction, also included a larger proportion of participants who attained an amyloid‐negative status compared to the global population (Table 2). The potential importance of achieving amyloid‐negative status to the clinical efficacy of gantenerumab is supported by an exploratory post hoc analysis of the global population of the GRADUATE studies, which suggested that amyloid negativity may be necessary in slowing the worsening of CDR‐SB.^16^ Notably, common baseline characteristics of those in the global population of the GRADUATE studies who attained an amyloid‐negative status included a lower body weight, lower amyloid load, and an earlier disease stage.^35^ Also, in line with this post hoc analysis in the GRADUATE studies, the Japanese participants of the GRADUATE studies who experienced cognitive‐, functional‐, and amyloid‐related benefits tended to have a lower baseline body weight, lower baseline amyloid load, and a higher percentage were enrolled with an MCI due to AD diagnosis at screening (i.e., at an earlier disease stage) compared to the global population of the GRADUATE studies.

In contrast, the reproducibility of favorable effects in slowing cognitive and functional decline in the Japanese participants in the GRADUATE studies was not confirmed in the Q2W double‐blind part of JP40959. Amyloid load reductions in gantenerumab versus placebo groups were observed across all three trials, but with varying magnitudes. The magnitude of amyloid load reduction in the Q2W double‐blind part of JP40959 was less than in Japanese participants in the GRADUATE studies, and comparable to that in the global population in the GRADUATE studies (Table S3, Figure S5). Notably, a lower proportion of JP40959 participants attained an amyloid‐negative status at week 104 (high‐dose gantenerumab, 9 of 24 [37.5%]) than Japanese participants in the GRADUATE studies at week 116 (gantenerumab, 8 of 11 [72.7%]). The global population of the GRADUATE studies had the lowest proportion of participants who attained an amyloid‐negative status at week 116 (gantenerumab 25 of 91 [27.5%]; Table S4). While baseline body weight in JP40959 was comparable to the Japanese participants in the GRADUATE studies, the JP40959 population had a lower percentage of people with MCI due to AD at screening (i.e., a later disease stage) and a slightly higher amyloid load (Table 1). Taken together, these results led to the hypothesis that both a low body weight and an earlier disease stage may play a role in achieving amyloid negativity, and consequently, slowing the worsening of cognition and function, but further investigations are needed to definitively confirm the hypothesis.

Evidence to support the relationship between lower baseline body weight and benefits on amyloid load reduction and cognitive and functional decline was observed when comparing the East Asian versus non‐East Asian participants in the GRADUATE studies.^26^ The association of low body weight with high amyloid load reduction is potentially due to increased gantenerumab exposure, as mean plasma gantenerumab concentrations (at least up to the occurrence of an irregular dose) were higher in the Japanese participants in the GRADUATE studies and high‐dose gantenerumab group of JP40959 than in the global population of the GRADUATE studies (Figure 4). However, the limited sample size, and imbalances in baseline disease characteristics and body weight, made it difficult to conclude the relationship between plasma gantenerumab exposure and treatment responses from these studies.

Additionally, a lower baseline amyloid load and milder disease severity have been previously suggested as contributing factors to cognitive and functional benefits and amyloid reduction in other studies.^36^, ^37^, ^38^ Participants with a lower amyloid load at baseline and a milder pathological severity may experience a greater anti‐Aβ monoclonal antibody treatment effect on amyloid load reduction. Furthermore, participants with a lower baseline amyloid load have fewer amyloid plaques to clear, potentially making it easier to achieve an amyloid‐negative status. The GRADUATE studies provided consistent trends favoring male versus female participants in cognitive and functional outcomes at Week 116, who not only had lower amyloid PET load but also had lower tau levels than female participants at baseline.^26^ Cognitive and functional outcomes in Japanese participants in the GRADUATE studies, excluding ADCS‐ADL total score, also provided better numerical trends in male participants (Table S10 in supporting information). However, the very small sample size of this subgroup makes it difficult to interpret the contribution of sex on treatment effect. While the further limited sample sizes for the Japanese participants in the GRADUATE amyloid PET substudy (for male participants, *n* = 6 in each group; for female participants, *n* = 8 in gantenerumab group and *n* = 10 in placebo group) need to be noted, the baseline amyloid load was comparable across sexes. In contrast, the change from baseline to week 116 in amyloid load in the gantenerumab group was higher for male participants (median/mean −83.30/−72.09 CL) than for female participants (median/mean −58.16/−60.39 CL; Table S10). This difference is potentially one of the reasons why better numerical trends in cognitive and functional outcomes were observed in male Japanese participants in the GRADUATE studies. For JP40959, the amyloid PET loads at baseline were higher than those of the Japanese participants in the GRADUATE studies regardless of sex (Table S10). This might have led to the different outcomes between the two Japanese populations; however, the extremely limited number of participants, especially with only four male participants in the placebo group (of whom only two reached week 104 prior to study termination by the sponsor), made it impossible to further explore the cause of the different outcomes between the two Japanese populations using just the existing data. In addition, due to the lack of tau level assessments for the two Japanese populations, further discussion involving baseline tau levels was not feasible.

Aside from baseline body weight, amyloid load, and disease severity, other factors might have impacted amyloid reduction and cognitive function. One of the reasons for the lower amyloid reduction in JP40959 versus the Japanese participants in the GRADUATE studies was considered to be the shorter, protocol‐specified, double‐blind treatment period (104 weeks in JP40959 vs. 116 weeks in the GRADUATE studies). As a result of receiving gantenerumab beyond 104 weeks of the double‐blind treatment period in the high‐dose gantenerumab group of JP40959, the proportion of participants with amyloid‐negative status at week 158 (i.e., OLE week 52), increased from week 104 (Table S4) and was comparable to that at week 116 of the Japanese participants in the GRADUATE studies. The comparability of these results is limited by the small number of participants with amyloid PET data at those time points. Furthermore, the worsening of CDR‐SB in JP40959 was milder in the placebo group than in the high‐ and low‐dose gantenerumab groups, which may be attributed to the lower baseline CDR‐SB. However, despite anti‐Aβ therapies typically having a better treatment response in *APOE* ε4 carriers,^39^ subgroup analyses in the GRADUATE studies showed that *APOE* ε4 carrier status, a genetic risk factor for AD,^40^ did not influence treatment responses. The lack of treatment response observed in JP40959 may minimally be attributed to the lack of homozygous *APOE* ε4 carriers in the placebo group of JP40959. Also, as the previously reported results of sensitivity analysis in the global population of the GRADUATE studies^16^ confirmed the robustness for the “missing at random” (MAR) assumption, the impact of MAR assumption in Japanese subjects in GRADUATE and JP40959, in which the proportions of withdrawals due to non‐administrative reasons (except for “study terminated by sponsor”) were approximately half of those for the global population of the GRADUATE studies, was expected to be smaller compared to the global population.

Generally, gantenerumab was well tolerated in the Japanese participants in the GRADUATE studies and JP40959. Overall, the incidence of AEs between the Japanese participants in the GRADUATE studies and JP40959 was comparable, and consistent with the safety profile seen in the overall of the GRADUATE studies and previous studies of gantenerumab.^16^, ^41^ As expected, based on previous studies of gantenerumab,^16^, ^41^ the incidence of ARIA was higher in the gantenerumab versus placebo groups. The incidence of ARIA in all populations evaluated here was comparable. Despite *APOE* ε4 carriers having a greater risk for developing anti‐Aβ therapy–induced ARIA,^42^ JP40959 having a smaller sample size with a higher potential for variance showed that ARIA rates were similar between homozygous and heterozygous *APOE* ε4 carriers, and non‐carriers.

Mean plasma gantenerumab concentrations in the Q1W open‐label part of JP40959 were lower than in the Q2W double‐blind, high‐dose gantenerumab group. It should be noted that this difference was not derived from imbalance in dose intensity between both dose groups because the data points collected after irregular dosing were excluded from data summarization. The sample sizes of dose groups were too small to explore the reason for this difference in plasma gantenerumab concentration between regimens with various dosing frequency.

The results of, and comparisons between, these studies had several limitations, and consequently should be interpreted with caution. JP40959 had a limited sample size and as a result, limited generalizability, increased the risk of random variability, and made it difficult to adequately compare differences with the GRADUATE studies, particularly the effect of gantenerumab on cognition and function. Additionally, JP40959 was terminated early, prior to the analysis of prespecified endpoint at week 104, which lowered the precision of estimation, possibly increasing the variability. Furthermore, it should be noted that the analysis for subgroup with limited size in study without considering subgroup factors at randomization is unlikely to definitively ensure the comparability between treatment groups within the subgroups, and cross‐trial comparisons even with similar trial designs provide no assurance of comparability across trials.

In summary, slowing of cognitive and functional decline and amyloid reduction were greater in Japanese participants in the GRADUATE studies than in the global population of the GRADUATE studies and JP40959. The results of these studies could have been related to imbalances in baseline body weight, amyloid load, and disease severity between the studies. Further investigation of the relationship between cognitive and functional outcomes and amyloid‐load reduction is required considering the limited sample size and inconsistency in treatment effect observed between Japanese participants in the GRADUATE studies and JP40959.

## CONFLICT OF INTEREST STATEMENT

Takashi Asada has received consulting fees and honoraria from Chugai Pharmaceutical Co., Ltd. Angeliki Thanasopoulou, Paul Delmar, and Jakub Wojtowicz are full‐time employees of and shareholder in F. Hoffmann‐La Roche Ltd. Janice Smith is an employee of Roche Products Ltd and shareholder in F. Hoffmann‐La Roche Ltd. Yasumasa Yoshiyama, Katsunori Yokoi, Chigusa Watanabe, and Mitsuhiro Isozaki have nothing to disclose. Ryoto Ozaki and Takaaki Ishida are employees of Chugai Pharmaceutical Co., Ltd. Hironori Tatsuda is an employee of and shareholder in Chugai Pharmaceutical Co., Ltd. Akira Tamaoka has received consulting fees from Chugai Pharmaceutical Co., Ltd. Author disclosures are available in the supporting information.

## CONSENT STATEMENT

The trials were conducted in accordance with the International Council for Harmonisation E6 Guideline for Good Clinical Practice and the principles of the Declaration of Helsinki, as well as the laws where the research was conducted. The protocol and any subsequent amendments were approved by the relevant institutional review board or ethics committee and by regulatory authorities. All trial participants provided written informed consent prior to study enrollment.

## Supporting information



Supporting Information

Supporting Information

## Data Availability

For the GRADUATE studies, qualified researchers may request access to individual participant‐level clinical data through a data request platform. At the time of writing, this request platform is Vivli (https://vivli.org/ourmember/roche/). For up‐to‐date details on Roche's Global Policy on the Sharing of Clinical Information and how to request access to related clinical study documents, see: https://go.roche.com/data_sharing. Anonymized records for individual participants across more than one data source external to Roche cannot, and should not, be linked due to a potential increase in risk of patient re‐identification. For the JP40959 study, qualified researchers may request access to individual patient‐level data through the clinical study data request platform (www.clinicalstudydatarequest.com). For further details on Chugai's Data Sharing Policy and how to request access to related clinical study documents, see: www.chugai‐pharm.co.jp/english/profile/rd/ctds_request.html.
