# Supplementary material for: Japanese participant data from three gantenerumab trials in early Alzheimer's disease
Source: Alzheimers Dement. 2025 Apr 29;21(4):e70192. doi: 10.1002/alz.70192 (PMC12040720; doi:10.1002/alz.70192)
Supplement: Supplementary file 2 — Supporting Information [file ALZ-21-e70192-s002.docx]

**Supplementary materials for:** **Japanese participant data from three gantenerumab trials in early Alzheimer’s disease**

**Contents**

[Clinical investigators and sites 3](#_Toc191550136)

[Japanese participants in GRADUATE I 3](#_Toc191550137)

[Japanese participants in GRADUATE II 3](#_Toc191550138)

[JP40959 4](#_Toc191550139)

[Supplementary methods 6](#_Toc191550140)

[Study design for Q1W open-label part and OLE period of JP40959 6](#_Toc191550141)

[Supplementary results 8](#_Toc191550142)

[Trial participant population and drug exposure for OLE period and Q1W open-label part of JP40959 8](#_Toc191550143)

[Effect of gantenerumab on amyloid load as measured by PET for Q1W open-label part and the OLE period of JP40959 9](#_Toc191550144)

[Supplementary tables and figures 10](#_Toc191550145)

[**SUPPLEMENTARY TABLE 1** Baseline demographics and characteristics of the Q1W open-label participants in JP40959 10](#_Toc191550146)

[**SUPPLEMENTARY TABLE 2** Treatment exposure in the GRADUATE studies and JP40959 12](#_Toc191550147)

[**SUPPLEMENTARY TABLE 3** Primary and secondary outcomes in the GRADUATE studies and the Q2W double-blind part of JP40959 14](#_Toc191550148)

[**SUPPLEMENTARY TABLE 4** Proportion of participants achieving amyloid positivity threshold by visit week in the GRADUATE studies and JP40959 17](#_Toc191550149)

[**SUPPLEMENTARY TABLE 5** Summary statistics of gantenerumab plasma concentrations in the GRADUATE studies and JP40959 20](#_Toc191550150)

[**SUPPLEMENTARY TABLE 6** Overview of safety after initial gantenerumab administration in the Japanese participants in the GRADUATE studies and JP40959 22](#_Toc191550151)

[**SUPPLEMENTARY TABLE 7** Most common adverse events with an incidence rate of ≥10% in any treatment group of the GRADUATE studies and the Q2W double-blind and Q1W open-label parts of JP40959 24](#_Toc191550152)

[**SUPPLEMENTARY TABLE 8** Most common adverse events with an incidence rate of ≥10% in any treatment group after initial gantenerumab administration in JP40959 26](#_Toc191550153)

[**SUPPLEMENTARY TABLE 9** Incidence of ARIA MRI findings in the Q2W OLE period and Q1W open-label part of JP40959 29](#_Toc191550154)

[**SUPPLEMENTARY TABLE 10** Primary and secondary outcomes in the GRADUATE studies and the Q2W double-blind part of JP40959 by sex 31](#_Toc191550155)

[**SUPPLEMENTARY FIGURE 1** Diagram of key study population included in the main manuscript 34](#_Toc191550156)

[**SUPPLEMENTARY FIGURE 2** Study design of the Q1W open-label part of JP40959 35](#_Toc191550157)

[**SUPPLEMENTARY FIGURE 3** Participant disposition of the Japanese participants in the GRADUATE studies (A), Q2W double-blind part and Q2W OLE period (B), Q1W open label (C) of JP40959 36](#_Toc191550158)

[(A) Participant disposition of the Japanese participants in the GRADUATE studies 36](#_Toc191550159)

[(B) Participant disposition of the Q2W double-blind part and Q2W OLE period of JP40959 37](#_Toc191550160)

[(C) Participant disposition of the Q1W open-label part of JP40959 38](#_Toc191550161)

[**SUPPLEMENTARY FIGURE 4** Adjusted mean change from baseline in cognitive and functional endpoints in the Q2W double-blind part of JP40959, and the Japanese and global populations in the GRADUATE studies 39](#_Toc191550162)

[(A) CDR-SB 39](#_Toc191550163)

[(B) ADAS-Cog13 40](#_Toc191550164)

[(C) ADCS-ADL total score 41](#_Toc191550165)

[(D) MMSE 42](#_Toc191550166)

[**SUPPLEMENTARY FIGURE 5** Adjusted mean change from baseline in amyloid load as measured by PET in the Q2W double-blind part of JP40959, and the Japanese and global populations in the GRADUATE studies 44](#_Toc191550167)

[**SUPPLEMENTARY FIGURE 6** Mean ± SD measured values of amyloid load on PET (CL) in participants who received gantenerumab in the Japanese participants in the GRADUATE studies and JP40959 46](#_Toc191550168)

# Clinical investigators and sites

## Japanese participants in GRADUATE I

Clinical sites: Koichi Kashiwado, Kashiwado Hospital; Tomokazu Obi, NHO Shizuoka Institute of Epilepsy and Neurological Disorders; Junya Kawada, Shonan Kamakura General Hospital; Kenichi Furihata, P-One Clinic; Chikako Kaneko, Southern Tohoku Medical Clinic; Shin Tanaka, Mishima Hospital; Kenichi Fujimoto, Jichiidai Station Brain Clinic; Soichiro Shimizu, Tokyo Medical University Hospital; Yasumasa Yoshiyama, Inage Neurology and Memory Clinic; Takashi Hata, Shizuoka City Shimizu Hospital; Kazuo Yamashiro, Juntendo University Urayasu Hospital; Masaharu Amagasa, Yamagata Tokushukai Hospital; Kazutomi Kanemaru, Tokyo Metropolitan Institute for Geriatrics and Gerontology; Masuhiro Sakata, National Center of Neurology and Psychiatry; Toru Kinoshita, Nozomi Memory Clinic; Takafumi Oga, Shinjuku Research Park Clinic;

Amyloid positron emission tomography (PET) imaging sites: Akitoshi Nakamori, Yuai Clinic; Weijey Ko, Yotsuya Medical Cube; Satoshi Ishikura, Tokyo Bay Advanced Medical and Makuhari Clinic; Ukihide Tateishi, Tokyo Medical and Dental University Hospital.

## Japanese participants in GRADUATE II

Clinical sites: Takao Mitsui, Tokushima National Hospital; Kenichi Shimada, Hyogo Prefectural Harima-Himeji General Medical Center; Manabu Hashimoto, Hizen Psychiatric Medical Center; Nobuya Kawabata, Yachiyo Hospital; Sadao Katayama, Katayama Medical Clinic; Chigusa Watanabe, Hiroshima-Nishi Medical Center; Katsunori Yokoi, National Center for Geriatrics and Gerontology; Tomotsugu Kaga, Nagoya Ekisaikai Hospital; Nobuyuki Takahashi, Nara City Hospital; Yoshio Tsuboi, Fukuoka University Hospital; Yutaka Matsui, Matsui Dietary and Dementia Clinic; Shinya Asayama, Tsukazaki Hospital; Yoshihiko Nishida, Itsuki Hospital; Makoto Izuta, Kishiwada Tokushukai Hospital; Yoshitomo Shirakashi, Uji Takeda Hospital; Yuko Wada, Rakuwakai Otowa Hospital; Nobutoshi Morimoto, Kagawa Prefectural Central Hospital;

Amyloid PET imaging sites: Seiki Hamada, MI Clinic; Munenobu Nogami, Kobe University Hospital; Yoshihiro Okumura, Okayama Kyokuto Hospital.

## JP40959

Clinical sites: Masakazu Sugino, Aino Hospital; Kentaro Deguchi, Okayama City Hospital; Naokazu Sasagasako, National Organization Omuta National Hospital; Tomoyuki Ono, Takesato Hospital; Hidenori Hattori, Tachikawa Hospital; Yasuhiro Yoshii, Koukan Clinic; Takenori Uozumi, Hospital of University of Occupational and Environmental Health, Japan; Misaki Toma, Tokyo Center Clinic; Hiroyuki Fukase, Clinical Research Hospital Tokyo; Chika Nishimura, Kurumi Clinic; Hiroshi Yamashita, Hiroshima City North Medical Center Asa Citizens Hospital; Akihiro Shindo, Mie University Hospital; Kenji Yoshiyama, Osaka University Hospital; Tomoyuki Kamata, Japanese Red Cross Musashino Hospital; Takashi Abe, Abe Neurological Clinic; Mitsuhiro Isozaki, Memory Clinic Toride; Kenichi Sakai, Minami-Okayama Medical Center; Yukari Imon, Imon Yukari Neurology Clinic;

Amyloid PET imaging sites: Seiki Hamada, MI Clinic; Ukihide Tateishi, Tokyo Medical and Dental University Hospital; Akitoshi Nakamori, Yuai Clinic; Masataka Nakagawa, Tokyo Bay Advanced Medical and Makuhari Clinic; Yoshihiro Okumura, Okayama Kyokuto Hospital; Munenobu Nogami, Kobe University Hospital.

# Supplementary methods

## Study design for Q1W open-label part and OLE period of JP40959

For the one dose every week (Q1W) open-label part, eligible participants received three doses of gantenerumab 120 mg every 4 weeks (Q4W), three doses of 255 mg Q4W, and six doses of 255 mg every 2 weeks (Q2W), followed by 255 mg Q1W from week 36 onwards up to 104 weeks (Supplementary Figure 2). The protocol-specified treatment duration at the target dose was 67 weeks in the Q1W open-label part. Participants were not required to meet any Free and Cued Selective Reminding Test (FCSRT) criteria.

Approximately 2 weeks after completion of the Q2W double-blind part or Q1W open-label part, participants had the opportunity to enroll in the Q2W open-label extension (OLE) or Q1W OLE periods, respectively, or complete a final assessment after a 48-week follow-up period. Participants were eligible for the Q2W OLE or Q1W OLE periods if they provided consent to participate in the respective OLE period, had not discontinued study treatment during the initial Q2W double-blind or Q1W open-label parts, and after the resolution of any amyloid-related imaging abnormalities (ARIA) magnetic resonance imaging (MRI) findings detected at week 104.

The protocol-specified treatment duration at the target dose was 67 weeks in Q1W open-label part. The algorithm ARIA management for the OLE period was based on the Bioclinica 5-point scale as a scale for the severity of ARIA.

The effect of gantenerumab on cognition and function in the Q1W open-label part could not be compared with the Q2W double-blind part of JP40959 and the GRADUATE studies due to the lack of a placebo control, and potential bias introduced by unblinding. The efficacy, safety, and pharmacokinetic analyses were assessed using summary statistics for the same endpoints as the Q2W double-blind part and were performed on all enrolled participants who received at least one dose of the study drug. No model analysis was performed, given the limited sample size and summary statistics were presented.

# Supplementary results

## Trial participant population and drug exposure for OLE period and Q1W open-label part of JP40959

Of the 42 participants who completed the Q2W double-blind part, 37 participants entered Q2W OLE period (high-dose gantenerumab, n = 20 [95.2%]; low-dose gantenerumab, n = 9 [75.0%]; placebo, n = 8 [88.9%]) (Supplementary Figure 3B). For the Q1W open-label part of JP40959, 11 participants were enrolled and treated. All of them discontinued the open-label treatment period due to study termination by the sponsor (Supplementary Figure 3C).

Participants in the Q1W open-label part had a milder disease severity of cognition and function at baseline, as measured by FCSRT, Clinical Dementia Rating – Sum of Boxes (CDR-SB), and Alzheimer’s Disease Assessment Scale – Cognitive Subscale 13 (ADAS-Cog13), versus the Q2W double-blind part of JP40959 (Supplementary Table 1). The baseline mean (standard deviation [SD]) amyloid PET load (Centiloid [CL]) in these participants (80.06 [21.32]) was comparable to the Japanese participants in the GRADUATE studies. For Q1W open-label part, the mean (SD) treatment duration at the target dose was 19.52 (6.44) weeks (Supplementary Table 2).

## Effect of gantenerumab on amyloid load as measured by PET for Q1W open-label part and the OLE period of JP40959

In the high- and low-dose gantenerumab groups, further amyloid load reduction was observed during the Q2W OLE period. In the Q1W open-label part, the mean (SD) was 52.04 (23.85) at week 52, in between high- and low-dose gantenerumab groups of the Q2W double-blind part at week 52 (Supplementary Figure 6). The proportions of participants with amyloid-negative status in the high- and low-dose gantenerumab groups increased in the Q2W OLE period following the Q2W double-blind part (75.0% [six of eight] and 80.0% [four of five] at OLE week 52). The number of participants with the amyloid-negative status at week 52 in Q1W open-label part and OLE period following placebo of the Q2W double-blind part of JP40959 were one (9.1%) and 0, respectively (Table 2).

# Supplementary tables and figures

## **SUPPLEMENTARY TABLE 1** Baseline demographics and characteristics of the Q1W open-label participants in JP40959

| **Characteristic** | **Q1W open-label part of JP40959 (*n* = 11)** |
| --- | --- |
| Age, years, mean (SD) | 69.3 (9.7) |
| Sex, Female, *n* (%) | 8 (72.7) |
| Race, Asian, *n* (%) | 11 (100) |
| Body weight, kg, mean (SD) | 51.78 (6.39) |
| BMI, kg/m^2^, mean (SD) | 21.68 (3.47) |
| *APOE* ε4 allele, n (%) |  |
| 0ɛ4 | 6 (54.5) |
| 1ɛ4 | 5 (45.5) |
| 2ɛ4 | 0 (0.0) |
| Use of AD medication at enrollment, *n* (%) | 5 (45.4) |
| AD diagnosis at screening, *n* (%) |  |
| MCI due to AD | 6 (54.5) |
| Mild AD dementia | 5 (45.5) |
| CDR-GS, *n* (%) |  |
| 0 | 1 (9.1)* |
| 0.5 | 10 (90.9) |
| 1 | 0 (0.0) |
| MMSE, mean (SD) | 24.4 (2.4) |
| FCSRT, mean (SD) |  |
| Free recall | 14.5 (8.1) |
| Cueing index | 0.55 (0.27) |
| CDR-SB, mean (SD) | 2.41 (1.04) |
| ADAS-Cog13, mean (SD) | 21.5 (8.6) |
| ADCS-ADL total score, mean (SD) | 69.9 (4.4) |
| Baseline amyloid PET as measure by PET, mean CL (SD) | 80.06 (21.32) |

NOTE. Assessment scoring ranges: CDR-GS, 0-3; MMSE, 0-30; FCSRT free recall, 0-48; FCSRT Cueing index, 0.0-1.0; CDR-SB, 0-18; ADAS-Cog13, 0-85; ADCS-ADL total score, 0-78.

*Baseline values; at screening all participants were confirmed to be eligible with CDR GS of 0.5 or 1.

AD, Alzheimer’s disease; ADAS-Cog 13, Alzheimer’s Disease Assessment Scale – Cognitive Subscale 13; ADCS-ADL, Alzheimer’s Disease Cooperative Study – Activities of Daily Living; *APOE* ε4, apolipoprotein E ε4 allele; BMI, body mass index; CDR-GS, Clinical Dementia Rating – Global Score; CDR-SB, Clinical Dementia Rating – Sum of Boxes; CL, Centiloid; FCSRT, Free and Cued Selective Reminding Test; MCI, mild cognitive impairment; MMSE, Mini-Mental State Examination; PET, positron emission tomography; Q1W, every week; SD, standard deviation.

## **SUPPLEMENTARY TABLE 2** Treatment exposure in the GRADUATE studies and JP40959

|  | **GRADUATE studies** | | | | | **JP40959** | | | | | | | |
| --- | --- | --- | --- | --- | --- | --- | --- | --- | --- | --- | --- | --- | --- |
|  | **Japanese participants** | | | **Global population** | | **Q2W double-blind part** | | | | **Q1W open-label part** | **Q2W OLE period** | | |
|  | **Gantenerumab (*n* = 59)** | **Placebo (*n* = 73)** | **Gantenerumab (*n* = 1004)** | | **Placebo (*n* = 955)** | **High-dose gantenerumab (*n* = 34)*** | **Low-dose gantenerumab (*n* = 17)** | **Placebo (*n* = 16)** | **Open-label gantenerumab (*n* = 11)** | | **High-dose gantenerumab (*n* = 20)^†^** | **Low-dose gantenerumab (*n* = 9)^†^** | **Placebo (*n* = 8)^†^** |
| Treatment duration, weeks, mean (SD)^‡^ | 102.6 (30.42) | 107.6 (20.39) | 96.6 (31.97) | | 101.8 (28.30) | 93.74 (18.84) | 87.78 (26.46) | 89.09 (25.27) | 57.05 (4.70) | | 34.75 (21.73) | 30.37 (24.29) | 32.79 (28.87) |
| Min-Max^‡^ | 0.1-114.6 | 8.0-114.6 | 0.1-116.9 | | 0.1-117.0 | 8.1-102.6 | 7.1-102.4 | 8.3-102.4 | 50.3-65.1 | | 0.1-68.1 | 2.3-62.1 | 0.1-64.1 |
| Treatment duration at target dose (1020 mg per 4 weeks), weeks, mean (SD) | 74.0 (12.52) | NA | 65.3 (21.46) | | NA | 57.19 (15.36) | NA | NA | 19.52 (6.44) | | 34.75 (21.73) | 31.43 (18.02) | 21.26 (8.47) |
| Min-Max^§^ | 22.1-79.0 | NA | 1.9-79.7 | | NA | 2.1-66.7 | NA | NA | 8.1-29.1 | | 0.1- 68.1 | 2.1-50.1 | 10.1-28.1 |
| Total cumulative dose, mg, mean (SD) | 19541.4 (6640.9) | NA | 16647.1 (7525.8) | | NA | 16679.6 (4758.2) | 6764.1 (2667.4) | NA | 7963.6 (1314.3) | | 8913.8 (5396.1) | 6516.7 (5371.8) | 4507.5 (4777.4) |
| Min-Max | 120-23055 | NA | 120-23055 | | NA | 360-19995 | 360-8625 | NA | 6225-9795 | | 510-17850 | 510-13260 | 120-10305 |

*The target dose of high-dose gantenerumab group in JP40959 is the same as in GRADUATE I and II.

^†^Assigned treatment group in the double-blind part of JP40959.

^‡^Treatment duration is the date of the last dose of study drug minus the date of the first dose plus one day in each part.

^§^The maximum protocol-specified treatment duration is 102 weeks in the Q2W double-blind part of JP40959, 103 weeks in the Q1W open-label part of JP40959, and 114 weeks in GRADUATE I and II, and the maximum for target dose is 66 weeks in the Q2W double-blind part of JP40959, 67 weeks in the Q1W open-label part of JP40959 and 78 weeks in GRADUATE I and II.

NA, not applicable; OLE, open-label extension; Q1W, every week; Q2W, every 2 weeks; SD, standard deviation.

## **SUPPLEMENTARY TABLE 3** Primary and secondary outcomes in the GRADUATE studies and the Q2W double-blind part of JP40959

|  | **GRADUATE studies** | | | | **JP40959** | | |
| --- | --- | --- | --- | --- | --- | --- | --- |
|  | **Japanese participants** | | **Global population** | | **Q2W double-blind part** | | |
| **Outcome** | **Gantenerumab (*n* = 59)** | **Placebo (*n* = 73)** | **Gantenerumab (*n* = 997)** | **Placebo (*n* = 962)** | **High-dose gantenerumab  (*n* = 34)** | **Low-dose gantenerumab  (*n* = 17)** | **Placebo (*n* = 16)** |
| Change from baseline in CDR-SB score at Week 104/116, adjusted mean (SE) | 1.83 (0.296) | 3.13 (0.265) | 3.02 (0.103) | 3.32 (0.103) | 2.05 (0.411) | 2.04 (0.576) | 1.65 (0.617) |
| Change from baseline in amyloid PET reduction at Week 104/116, adjusted mean (SE)* | –65.80 (3.593)* | 12.23 (3.401)* | –53.08 (2.001)^†^ | 8.84 (2.039)^†^ | –53.83 (3.494) | –39.40 (5.098) | 6.35 (5.221) |
| Change from baseline in ADAS-Cog13 score at Week 104/116, adjusted mean (SE) | 3.97 (1.057) | 6.97 (0.947) | 7.42 (0.339) | 8.77 (0.338) | 6.37 (1.247) | 8.58 (1.732) | 6.78 (1.843) |
| Change from baseline in ADCS-ADL total score at Week 104/116, adjusted mean (SE) | –4.43 (1.264) | –7.92 (1.134) | –9.77 (0.438) | –10.83 (0.438) | –4.83 (1.628) | –4.14 (2.287) | –3.79 (2.423) |
| Change from baseline in MMSE total score at Week 104/116, adjusted mean (SE) | –3.09 (0.636) | –4.53 (0.569) | –4.42 (0.161) | –4.84 (0.161) | –3.67 (0.832) | –4.19 (1.168) | –3.61 (1.236) |

NOTE. In the GRADUATE studies, the primary efficacy endpoint was the change from baseline to Week 116 in CDR–SB. In JP40959, the primary endpoint was the change from baseline to Week 104 in amyloid load as measured by PET SUVR; however, to compare the amyloid load on PET between JP40959 and the GRADUATE studies, the results were converted to CL units.

*A total of 30 Japanese participants in the GRADUATE studies, (gantenerumab group, n = 14; placebo group, n = 16) were enrolled in the amyloid PET substudy.

^†^A total of 308 participants in the global population of the GRADUATE studies (gantenerumab group, n = 160; placebo group, n = 148) were enrolled in the amyloid PET substudy.

ADAS-Cog13, Alzheimer's Disease Assessment Scale – Cognitive Subscale 13; ADCS-ADL, Alzheimer’s Disease Cooperative Study – Activities of Daily Living; CDR-SB, Clinical Dementia Rating – Sum of Boxes; CL, Centiloid; MMSE, Mini-Mental State Examination; PET, positron emission tomography; Q2W, every 2 weeks; SE, standard error; SUVR, standardized uptake value ratio.

**SUPPLEMENTARY TABLE 4** Proportion of participants achieving amyloid positivity threshold by visit week in the GRADUATE studies and JP40959

|  | **GRADUATE studies** | | | | **JP40959** | | | | |
| --- | --- | --- | --- | --- | --- | --- | --- | --- | --- |
|  | **Japanese participants*** | | **Global population**^†^ | |  | | | | |
|  | **Gantenerumab (*n* = 14)** | **Placebo (*n* = 16)** | **Gantenerumab  (*n* = 160)** | **Placebo (*n* = 148)** | **High-dose gantenerumab in Q2W double-blind and its OLE (*n* = 34)** | **Low-dose gantenerumab in Q2W double-blind and its OLE (*n* = 17)** | **Placebo in Q2W double-blind (*n* = 16)** | **OLE following placebo in Q2W double-blind (*n* = 8)** | **Q1W open-label gantenerumab  (*n* =11)** |
| Baseline |  |  |  |  |  |  |  |  |  |
| *n* | 14 | 16 | 160 | 148 | 34 | 17 | 16 | 8 | 11 |
| Baseline amyloid load as measured by PET, mean CL (SD) | 81.04 (22.39) | 79.56 (24.83) | 94.70 (27.04) | 94.23 (31.87) | 84.99 (23.40) | 85.23 (16.88) | 86.54 (22.12) | 93.45 (23.09) | 80.06 (21.32) |
| ≤ 24 CL, *n* (%) | 0 (0.0) | 0 (0.0) | 2 (1.3) | 3 (2.0) | 0 (0.0) | 0 (0.0) | 0 (0.0) | 0 (0.0) | 0 (0.0) |
| > 24 CL, *n* (%) | 14 (100) | 16 (100) | 158 (98.8) | 145 (98.0) | 34 (100) | 17 (100) | 16 (100) | 8 (100) | 11 (100) |
| Week 52 |  |  |  |  |  |  |  |  |  |
| *n* | 10 | 13 | 100 | 100 | 30 | 13 | 14 | 4 | 11 |
| ≤ 24 CL, *n* (%) | 0 (0.0) | 0 (0.0) | 2 (2.0) | 1 (1.0) | 2 (6.7) | 0 (0.0) | 0 (0.0) | 0 (0.0) | 1 (9.1) |
| > 24 CL, *n* (%) | 10 (100) | 13 (100) | 98 (98.0) | 99 (99.0) | 28 (93.3) | 13 (100) | 14 (100) | 4 (100) | 10 (90.9) |
| Week 104 |  |  |  |  |  |  |  |  |  |
| *n* | NE | NE | NE | NE | 24 | 12 | 10 | 0 | 0 |
| ≤ 24 CL, *n* (%) | NE | NE | NE | NE | 9 (37.5) | 1 (8.3) | 0 (0.0) | NE | NE |
| > 24 CL, *n* (%) | NE | NE | NE | NE | 15 (62.5) | 11 (91.7) | 10 (100) | NE | NE |
| Week 116 |  |  |  |  |  |  |  |  |  |
| *n* | 11 | 12 | 91 | 87 | NE | NE | NE | NE | NE |
| ≤ 24 CL, *n* (%) | 8 (72.7) | 0 (0.0) | 25 (27.5) | 1 (1.1) | NE | NE | NE | NE | NE |
| > 24 CL, *n* (%) | 3 (27.3) | 12 (100) | 66 (72.5) | 86 (98.9) | NE | NE | NE | NE | NE |
| Week 158^‡^ |  |  |  |  |  |  |  |  |  |
| *n* | NE | NE | NE | NE | 8 | 5 | 0 | 0 | 0 |
| ≤ 24 CL, *n*(%) | NE | NE | NE | NE | 6 (75.0) | 4 (80.0) | NE | NE | NE |
| > 24 CL, *n* (%) | NE | NE | NE | NE | 2 (25.0) | 1 (20.0) | NE | NE | NE |

NOTE. Amyloid positivity is defined as ≤ 24 CL.

*A total of 30 Japanese participants in the GRADUATE studies, (gantenerumab group, *n* = 14; placebo group, *n* = 16) were enrolled in the amyloid PET substudy.

^†^A total of 308 participants in the global population of the GRADUATE studies (gantenerumab group, *n* = 160; placebo group,
*n* = 148) were enrolled in the amyloid PET substudy.

^‡^The protocol-specified OLE day 1 is approximately 2 weeks after week 104 in JP40959. OLE week 52, when counted from week 0 as the starting point, becomes week 158.

Abbreviations: CL, Centiloid; NE, not evaluable; OLE, open-label extension; PET, positron emission tomography; Q1W, every week; Q2W, every 2 weeks; SD, standard deviation.

## **SUPPLEMENTARY TABLE 5** Summary statistics of gantenerumab plasma concentrations in the GRADUATE studies and JP40959

|  | **Gantenerumab plasma concentration, μg/mL, mean ± SD (*n*)** | | | | | |
| --- | --- | --- | --- | --- | --- | --- |
|  | **GRADUATE studies** | | **JP40959** | | | |
|  | **Japanese participants** | **Global population** |  |  |  |  |
| **Time point** | **Gantenerumab (*n* = 59)** | **Gantenerumab**  **(*n* = 957)** | **High-dose gantenerumab in Q2W double-blind and its OLE (*n* = 34)** | **Low-dose gantenerumab in Q2W double-blind and its OLE (*n* = 17)** | **OLE following placebo in Q2W double-blind (*n* = 8)** | **Q1W open label gantenerumab (*n* = 11)** |
| Day 1 | NC (1) | NC (32) | NC (1) | NC (0) | NA | 0.0117 ± 0.00913 (11) |
| Day 4 | 7.19 ± 2.14 (59) | 11.4 ± 170 (943) | NA | NA | NA | NA |
| Week 1 | NA | NA | 8.23 ± 3.32 (34) | 9.33 ± 2.53 (17) | NA | 7.47 ± 2.75 (11) |
| Week 24 | 13.9 ± 10.3 (53) | 26.3 ± 405 (805) | 15.5 ± 6.78 (28) | 14.4 ± 4.67 (15) | NA | 13.3 ± 9.83 (10) |
| Week 41 | 96.7 ± 40.1 (47) | 79.7 ± 244 (498) | 83.4 ± 34.2 (21) | 39.7 ± 20.7 (12) | NA | 50.8 ± 20.8 (9) |
| Week 52 | 76.8 ± 32.4 (33) | 72.9 ± 119 (364) | 84.8 ± 47.3 (21) | 22.4 ± 13.2 (12) | NA | 46.3 ± 18.7 (7) |
| Week 76 | 86.1 ± 43.7 (29) | 66.0 ± 37.0 (243) | 81.7 ± 46.2 (16) | 23.4 ± 12.5 (10) | NA | NA |
| Week 103 | 98.8 ± 31.8 (24) | 78.9 ± 37.6 (170) | 93.8 ± 43.9 (11) | 19.9 ± 7.92 (9) | NA | NA |
| Week 115 | 93.0 ± 29.4 (24) | 84.8 ± 40.3 (142) | NA | NA | NA | NA |
| OLE day 1 | NA | NA | 50.8 ± 34.9 (11) | 10.5 ± 4.54 (9) | NC (2) | NA |
| OLE week 1 | NA | NA | 70.2 ± 38.5 (11) | 40.1 ± 18.9 (8) | 5.53 ± 2.58 (6) | NA |
| OLE week 24 | NA | NA | 94.0 ± 49.9 (7) | 70.3 ± 28.1 (4) | 16.7 ± 6.19 (4) | NA |
| OLE week 41 | NA | NA | 71.7 ± 36.2 (5) | 78.0 ± NC (1) | 77.6 ± 34.6 (4) | NA |
| OLE week 52 | NA | NA | 48.0 ± 14.3 (5) | 59.1 ± NC (1) | 51.3 ± 4.67 (2) | NA |

All data points collected after at least one irregular dose were excluded from this analysis. If more than half of the measured values are below the limit of quantification (0.500 ng/mL) at a sampling time point, the summary statistics other than *n* are shown as NC.

NA, not applicable; NC, not calculated; OLE, open-label extension; Q1W, every week; Q2W, every 2 weeks; SD, standard deviation.

## **SUPPLEMENTARY TABLE 6** Overview of safety after initial gantenerumab administration in the Japanese participants in the GRADUATE studies and JP40959

|  | **Japanese participants in the GRADUATE studies** | **JP40959** | | | |
| --- | --- | --- | --- | --- | --- |
|  | **Gantenerumab (*n* = 59)** | **High-dose gantenerumab in Q2W double-blind and its OLE (*n* = 34)** | **Low-dose gantenerumab in Q2W double-blind and its OLE (*n* = 17)** | **OLE following placebo in Q2W double-blind  (*n* = 8)** | **Q1W open-label gantenerumab (*n* = 11)** |
| Participants who experienced an AE, *n* (%) | 54 (91.5) | 31 (91.2) | 16 (94.1) | 5 (62.5) | 9 (81.8) |
| Participants who experienced an SAE, n (%) | 4 (6.8) | 8 (23.5) | 4 (23.5) | 1 (12.5) | 1 (9.1) |
| Participants permanently discontinuing treatment due to an AE, *n* (%) | 6 (10.2) | 0 (0.0) | 1 (5.9) | 0 (0.0) | 0 (0.0) |
| Participants who experienced an AE with a fatal outcome, *n* (%) | 0 (0.0) | 0 (0.0) | 1 (5.9) | 0 (0.0) | 0 (0.0) |
| Participants who experienced an AE related to study treatment, *n* (%) | 22 (37.3) | 14 (41.2) | 8 (47.1) | 1 (12.5) | 4 (36.4) |
| ARIA-E, *n* (%)* | 12 (20.3) | 6 (17.6) | 5 (29.4) | 0 (0.0) | 1 (9.1) |
| ARIA-H, *n* (%)^†^ | 4 (6.8) | 1 (2.9) | 4 (23.5) | 0 (0.0) | 1 (9.1) |
| ISR, *n* (%) | 4 (6.8) | 5 (14.7) | 3 (17.6) | 0 (0.0) | 2 (18.2) |

*ARIA-E includes events "Amyloid Related Imaging Abnormality-Oedema/Effusion", "Vasogenic Cerebral Oedema", and “Brain Oedema". ARIA-E had to be reported as an AE if it was symptomatic, led to dosing intervention, or was otherwise considered clinically significant by the investigator.

^†^ARIA-H includes events "Amyloid Related Imaging Abnormality-Microhaemorrhages And Haemosiderin Deposits", "Cerebral Microhaemorrhage", "Cerebral Haemosiderin Deposition", "Cerebellar Microhaemorrhage", and "Brain Stem Microhaemorrhage". ARIA-H had to be reported as an AE if it led to dosing intervention or was otherwise considered clinically significant by the investigator.

AE, adverse event; ARIA-E, amyloid-related imaging abnormalities – edema; ARIA-H, amyloid-related imaging abnormalities – hemosiderosis; ISR, injection site reaction; OLE, open-label extension; Q1W, every week; Q2W, every 2 weeks; SAE, serious adverse event.

## **SUPPLEMENTARY TABLE 7** Most common adverse events with an incidence rate of ≥10% in any treatment group of the GRADUATE studies and the Q2W double-blind and Q1W open-label parts of JP40959

|  | **Japanese participants in the GRADUATE studies** | | **Global population in the GRADUATE studies** | | **Q2W double-blind part of JP40959** | | | **Q1W open label gantenerumab**  **(*n* = 11)** |
| --- | --- | --- | --- | --- | --- | --- | --- | --- |
| **Participants, n (%)** | **Gantenerumab  (*n* = 59)** | **Placebo  (*n* = 73)** | **Gantenerumab  (*n* = 1004)** | **Placebo  (*n* = 955)** | **High-dose gantenerumab  (*n* = 34)** | **Low-dose gantenerumab  (*n* = 17)** | **Placebo**  **(*n* = 16)** |  |
| ARIA-E* | 12 (20.3) | 3 (4.1) | 219 (21.8) | 17 (1.8) | 6 (17.6) | 3 (17.6) | 1 (6.3) | 1 (9.1) |
| Back pain | 2 (3.4) | 5 (6.8) | 72 (7.2) | 72 (7.5) | 5 (14.7) | 2 (11.8) | 1 (6.3) | 1 (9.1) |
| Nasopharyngitis | 9 (15.3) | 14 (19.2) | 91 (9.1) | 82 (8.6) | 4 (11.8) | 2 (11.8) | 3 (18.8) | 0 (0.0) |
| Arthralgia | 6 (10.2) | 4 (5.5) | 76 (7.6) | 74 (7.7) | 3 (8.8) | 2 (11.8) | 2 (12.5) | 0 (0.0) |
| Fall | 4 (6.8) | 9 (12.3) | 113 (11.3) | 114 (11.9) | 6 (17.6) | 0 (0.0) | 1 (6.3) | 0 (0.0) |
| Hypertension | 2 (3.4) | 6 (8.2) | 75 (7.5) | 70 (7.3) | 3 (8.8) | 2 (11.8) | 1 (6.3) | 1 (9.1) |
| ARIA-H^†^ | 2 (3.4) | 1 (1.4) | 68 (6.8) | 6 (0.6) | 1 (2.9) | 3 (17.6) | 1 (6.3) | 1 (9.1) |
| Contusion | 1 (1.7) | 8 (11.0) | 33 (3.3) | 28 (2.9) | 6 (17.6) | 0 (0.0) | 0 (0.0) | 0 (0.0) |
| Dizziness | 1 (1.7) | 2 (2.7) | 84 (8.4) | 70 (7.3) | 2 (5.9) | 2 (11.8) | 2 (12.5) | 0 (0.0) |
| Cataract | 2 (3.4) | 4 (5.5) | 18 (1.8) | 20 (2.1) | 1 (2.9) | 2 (11.8) | 1 (6.3) | 1 (9.1) |
| Injection-related reaction | 0 (0.0) | 0 (0.0) | 0 (0.0) | 0 (0.0) | 4 (11.8) | 0 (0.0) | 0 (0.0) | 1 (9.1) |
| Vomiting | 2 (3.4) | 2 (2.7) | 49 (4.9) | 30 (3.1) | 2 (5.9) | 1 (5.9) | 2 (12.5) | 0 (0.0) |
| Diarrhoea | 1 (1.7) | 3 (4.1) | 72 (7.2) | 51 (5.3) | 1 (2.9) | 3 (17.6) | 0 (0.0) | 0 (0.0) |
| Injection site reaction | 4 (6.8) | 2 (2.7) | 169 (16.8) | 74 (7.7) | 0 (0.0) | 2 (11.8) | 0 (0.0) | 2 (18.2) |
| Rash | 2 (3.4) | 2 (2.7) | 26 (2.6) | 28 (2.9) | 2 (5.9) | 0 (0.0) | 0 (0.0) | 2 (18.2) |
| Osteoarthritis | 2 (3.4) | 6 (8.2) | 25 (2.5) | 30 (3.1) | 0 (0.0) | 2 (11.8) | 0 (0.0) | 0 (0.0) |
| Sinusitis | 1 (1.7) | 1 (1.4) | 10 (1.0) | 8 (0.8) | 0 (0.0) | 0 (0.0) | 2 (12.5) | 0 (0.0) |
| Headache | 2 (3.4) | 2 (2.7) | 124 (12.4) | 93 (9.7) | 1 (2.9) | 1 (5.9) | 0 (0.0) | 1 (9.1) |

*ARIA-E includes events "Amyloid Related Imaging Abnormality-Oedema/Effusion", "Vasogenic Cerebral Oedema", and “Brain Oedema". ARIA-E had to be reported as an AE if it was symptomatic, led to dosing intervention, or was otherwise considered clinically significant by the investigator.

^†^ARIA-H includes events "Amyloid Related Imaging Abnormality-Microhaemorrhages And Haemosiderin Deposits", "Cerebral Microhaemorrhage", "Cerebral Haemosiderin Deposition", "Cerebellar Microhaemorrhage", and "Brain Stem Microhaemorrhage". ARIA-H had to be reported as an AE if it led to dosing intervention or was otherwise considered clinically significant by the investigator.

AE, adverse event; ARIA-E, amyloid-related imaging abnormalities – edema; ARIA-H, amyloid-related imaging abnormalities – hemosiderosis; Q1W, every week; Q2W, every 2 weeks.

## **SUPPLEMENTARY TABLE 8** Most common adverse events with an incidence rate of ≥10% in any treatment group after initial gantenerumab administration in JP40959

| **Participants, *n* (%)** | **High-dose gantenerumab in Q2W double-blind and its OLE (*n* = 34)** | **Low-dose gantenerumab in Q2W double-blind and its OLE (*n* = 17)** | **OLE following placebo in Q2W double-blind (*n* = 8)** |
| --- | --- | --- | --- |
| At least one AE | 31 (91.2) | 16 (94.1) | 5 (62.5) |
| ARIA-E* | 6 (17.6) | 5 (29.4) | 0 (0.0) |
| Back pain | 5 (14.7) | 2 (11.8) | 0 (0.0) |
| Contusion | 6 (17.6) | 1 (5.9) | 1 (12.5) |
| Fall | 7 (20.6) | 0 (0.0) | 1 (12.5) |
| Nasopharyngitis | 6 (17.6) | 2 (11.8) | 0 (0.0) |
| Injection-related reaction | 5 (14.7) | 1 (5.9) | 0 (0.0) |
| Arthralgia | 4 (11.8) | 2 (11.8) | 0 (0.0) |
| Hypertension | 3 (8.8) | 2 (11.8) | 0 (0.0) |
| ARIA-H^†^ | 1 (2.9) | 3 (17.6) | 0 (0.0) |
| Corona virus infection | 4 (11.8) | 0 (0.0) | 0 (0.0) |
| Dizziness | 2 (5.9) | 3 (17.6) | 0 (0.0) |
| Cataract | 1 (2.9) | 2 (11.8) | 0 (0.0) |
| Diarrhoea | 1 (2.9) | 3 (17.6) | 0 (0.0) |
| Injection site reaction | 0 (0.0) | 2 (11.8) | 0 (0.0) |
| Pain in extremity | 2 (5.9) | 1 (5.9) | 1 (12.5) |
| Anxiety | 0 (0.0) | 0 (0.0) | 2 (25.0) |
| Eczema | 1 (2.9) | 2 (11.8) | 0 (0.0) |
| Vaccination site pain | 2 (5.9) | 0 (0.0) | 1 (12.5) |
| Dermatitis | 1 (2.9) | 0 (0.0) | 1 (12.5) |
| Neck pain | 1 (2.9) | 0 (0.0) | 1 (12.5) |
| Osteoarthritis | 0 (0.0) | 2 (11.8) | 0 (0.0) |
| Urticaria | 1 (2.9) | 0 (0.0) | 1 (12.5) |
| Crowned dens syndrome | 0 (0.0) | 0 (0.0) | 1 (12.5) |
| Gastroenteritis | 0 (0.0) | 0 (0.0) | 1 (12.5) |
| Intracranial aneurysm | 0 (0.0) | 0 (0.0) | 1 (12.5) |
| Neuropathy peripheral | 0 (0.0) | 0 (0.0) | 1 (12.5) |
| Tension headache | 0 (0.0) | 0 (0.0) | 1 (12.5) |

*ARIA-E includes events "Amyloid Related Imaging Abnormality-Oedema/Effusion", "Vasogenic Cerebral Oedema", and “Brain Oedema". ARIA-E had to be reported as an AE if it was symptomatic, led to dosing intervention, or was otherwise considered clinically significant by the investigator.

^†^ARIA-H includes events "Amyloid Related Imaging Abnormality-Microhaemorrhages And Haemosiderin Deposits", "Cerebral Microhaemorrhage", "Cerebral Haemosiderin Deposition", "Cerebellar Microhaemorrhage", and "Brain Stem Microhaemorrhage". ARIA-H had to be reported as an AE if it led to dosing intervention or was otherwise considered clinically significant by the investigator.

AE, adverse event; ARIA-E, amyloid-related imaging abnormalities – edema; ARIA-H, amyloid-related imaging abnormalities – hemosiderosis; OLE, open-label extension; Q2W, every 2 weeks.

## **SUPPLEMENTARY TABLE 9** Incidence of ARIA MRI findings in the Q2W OLE period and Q1W open-label part of JP40959

|  | **Q2W OLE period of JP40959** | | | **Q1W open-label part of JP40959** |
| --- | --- | --- | --- | --- |
| **ARIA incidence** | **High-dose gantenerumab in Q2W double-blind (*N* = 20)** | **Low dose gantenerumab in Q2W double-blind (*N* = 9)** | **Placebo in Q2W double-blind (*N* = 8)** | **Gantenerumab  (*N* = 11)** |
| Incidence of ARIA-E, *n/N* (%) | 3/20 (15.0) | 2/9 (22.2) | 0/8 (0.0) | 1/11 (9.1) |
| ARIA-E by *APOE* *ε4* genotype, n/total *n* (%) |  |  |  |  |
| *0 ε4* | 1/6 (16.7) | 0/2 (0.0) | 0/1 (0.0) | 1/6 (16.7) |
| *1 ε4* | 2/9 (22.2) | 1/6 (16.7) | 0/7 (0.0) | 0/5 (0.0) |
| *2 ε4* | 0/5 (0.0) | 1/1 (100) | 0/0 (0.0) | 0/0 (0.0) |
| Recurrent ARIA-E, *n/N* (%) | 1/20 (5.0) | 0/9 (0.0) | 0/8 (0.0) | 0/11 (0.0) |
| Symptomatic ARIA-E, *n/N* (%)* | 0/20 (0.0) | 0/9 (0.0) | 0/8 (0.0) | 0/11 (0.0) |
| Serious symptomatic ARIA-E, *n/N* (%)^†^ | 0/20 (0.0) | 0/9 (0.0) | 0/8 (0.0) | 0/11 (0.0) |
| Most severe ARIA-E (BGTS), mean (SD)^‡^ | - | - | - | 3.0 (NE) |
| Most severe ARIA-E (Bioclinica), mean (SD)^‡^ | 2.3 (1.5) | 3.0 (1.4) | NE (NE) | - |
| Overall incidence of ARIA-H^§^ | 0 (0.0) | 2 (22.2) | 0 (0.0) | 2 (18.2) |
| Incidence of ARIA-H concurrent with ARIA-E^¶^ | 0 (0.0) | 1 (11.1) | 0 (0.0) | 1 (9.1) |
| Incidence of isolated ARIA-H^#^ | 0 (0.0) | 1 (11.1) | 0 (0.0) | 1 (9.1) |

NOTE. Data for participants who underwent MRI after baseline are shown; those who discontinued the trial before assessment are not included.

*Symptomatic ARIA-E is defined as, for Q2W OLE period, the onset or worsening of CNS symptoms with a relationship to the MRI findings of the ARIA-E in the judgement of the investigator, for Q1W open-label part, ARIA-E temporally associated with CNS symptoms.

^†^Either the ARIA-E or CNS symptom(s) were reported as a serious AE.

^‡^In the Q2W double-blind and Q1W open-label, BGTS was used as a scale to measure ARIA severity. In the OLE periods of JP40959 the Bioclinica 5-point scale was used to measure ARIA severity for its simplicity.

^§^The counts represent cumulative post-baseline findings, excluding any corresponding ARIA-H at baseline in the Q2W OLE period and Q1W open-label part.

^¶^Concurrence is defined as temporal co-occurrence, with new ARIA-H MRI findings detected at the same time as emerging or ongoing ARIA-E MRI findings.

^#^Participants who did not develop incident ARIA-E during the Q2W OLE period and Q1W open-label part.

AE, adverse event; *APOE* ε4, apolipoprotein ε4 allele; ARIA, amyloid-related imaging abnormalities; ARIA-E, amyloid-related imaging abnormalities – edema; ARIA-H, amyloid related imaging abnormalities– hemosiderosis; BGTS, Barkhof Grand Total Scale; CNS, central nervous system; MRI, magnetic resonance imaging; NE, not evaluable; OLE, open-label extension; Q1W, every week; Q2W, every 2 weeks; SD, standard deviation.

## **SUPPLEMENTARY TABLE 10** Primary and secondary outcomes in the GRADUATE studies and the Q2W double-blind part of JP40959 by sex

|  | **GRADUATE studies** | | | | **JP40959** | | |
| --- | --- | --- | --- | --- | --- | --- | --- |
|  | **Japanese participants** | | **Global population** | | **Q2W double-blind part** | | |
| **Outcome** | **Gantenerumab (*n* = 59)**  **M: *n* = 25**  **F: *n* = 34** | **Placebo  (*n* = 73)**  **M: *n* = 31**  **F: *n* = 42** | **Gantenerumab (*n* = 997)**  **M: *n* = 419**  **F: *n* = 578** | **Placebo  (*n* = 962)**  **M: *n* = 422**  **F: *n* = 540** | **High-dose gantenerumab (*n* = 34)**  **M: *n* = 15**  **F: *n* = 19** | **Low-dose gantenerumab (*n* = 17)**  **M: *n* = 5**  **F: *n* = 12** | **Placebo  (*n* = 16)**  **M: *n* = 4**  **F: *n* = 12** |
| Baseline amyloid load as measured by PET, mean CL (SD) | M: 83.89 (32.54)*  F: 78.91 (12.76)* | M: 74.77 (17.51)*  F: 82.44 (28.86)* | M: 90.10 (28.03)^†^  F: 98.96 (25.53)^†^ | M: 89.31 (32.29)^†^  F: 98.65 (31.03)^†^ | M: 84.65 (19.23)  F: 85.25 (26.77) | M: 82.27 (9.84)  F: 86.47 (19.32) | M: 85.63 (26.77)  F: 86.84 (21.70) |
| Change from baseline in CDR-SB score at Week 104/116, adjusted mean (SE) | M: 1.47 (0.456)  F: 2.08 (0.378) | M: 3.29 (0.412)  F: 3.00 (0.338) | M: 2.75 (0.160)  F: 3.22 (0.135) | M: 3.27 (0.154)  F: 3.36 (0.138) | M: 2.59 (0.839)  F: 1.78 (0.451) | M: 1.36 (1.508)  F: 2.24 (0.555) | M: 1.99 (1.659)  F: 1.33 (0.584) |
| Change from baseline in amyloid PET reduction at Week 104/116, adjusted mean (SE) | M: –72.09 (7.441)*  F: –60.39 (3.693)* | M: 9.87 (7.778)*  F: 11.72 (3.423)* | M: –49.90 (2.741)^†^  F: –56.16 (3.012)^†^ | M: 10.62 (2.831)^†^  F: 7.21 (3.019)^†^ | M: –48.62 (6.153)  F: -56.48 (4.604) | M: –33.89 (10.586)  F: -42.58 (6.294) | M: 7.95 (15.322)  F: 6.58 (5.577) |
| Change from baseline in ADAS-Cog13 score at Week 104/116, adjusted mean (SE) | M: 3.58 (1.439)  F: 4.34 (1.531) | M: 7.50 (1.288)  F: 6.56 (1.370) | M: 7.19 (0.550)  F: 7.57 (0.428) | M: 9.34 (0.533)  F: 8.30 (0.437) | M: 9.34 (2.248)  F: 3.73 (1.410) | M: 8.79 (3.755)  F: 8.45 (1.733) | M: 0.78 (4.578)  F: 8.10 (1.811) |
| Change from baseline in ADCS-ADL total score at Week 104/116, adjusted mean (SE) | M: –4.86 (1.432)  F: –4.01 (1.907) | M: –7.99 (1.295)  F: –7.98 (1.710) | M: –9.44 (0.699)  F: –10.02 (0.564) | M: –11.59 (0.676)  F: –10.21 (0.579) | M: –6.03 (2.927)  F: –3.94 (1.867) | M: –5.34 (4.964)  F: –4.62 (2.322) | M: 3.41 (6.083)  F: –5.46 (2.420) |
| Change from baseline in MMSE total score at Week 104/116, adjusted mean (SE) | M: –2.52 (0.882)  F: –3.43 (0.902) | M: –4.77 (0.791)  F: –4.38 (0.807) | M: –4.13 (0.254)  F: –4.61 (0.208) | M: –5.02 (0.245)  F: –4.70 (0.212) | M: –4.98 (1.754)  F: –2.44 (0.756) | M: –4.89 (3.119)  F: –4.09 (0.937) | M: –0.18 (3.696)  F: –4.20 (0.966) |

NOTE. In the GRADUATE studies, the primary efficacy endpoint was the change from baseline to Week 116 in CDR–SB. In JP40959, the primary endpoint was the change from baseline to Week 104 in amyloid load as measured by PET SUVR; however, to compare the amyloid load on PET between JP40959 and the GRADUATE studies, the results were converted to CL units.

*A total of 30 Japanese participants in the GRADUATE studies, (gantenerumab group, n = 14 [M, n = 6; F, n = 8]; placebo group, n = 16 [M, n = 6; F, n = 10]) were enrolled in the amyloid PET substudy.

^†^A total of 308 participants in the global population of the GRADUATE studies (gantenerumab group, n = 160 [M, n= 77; F, n = 83]; placebo group, n = 148 [M, n = 70; F, n = 78]) were enrolled in the amyloid PET substudy.

ADAS-Cog13, Alzheimer's Disease Assessment Scale – Cognitive Subscale 13; ADCS-ADL; Alzheimer’s Disease Cooperative Study – Activities of Daily Living; CDR-SB, Clinical Dementia Rating – Sum of Boxes; CL, Centiloid; F, female; M, male; MMSE, Mini-Mental State Examination; PET, positron emission tomography; Q2W, every 2 weeks; SE, standard error; SUVR, standardized uptake value ratio.

## **SUPPLEMENTARY FIGURE 1** Diagram of key study population included in the main manuscript


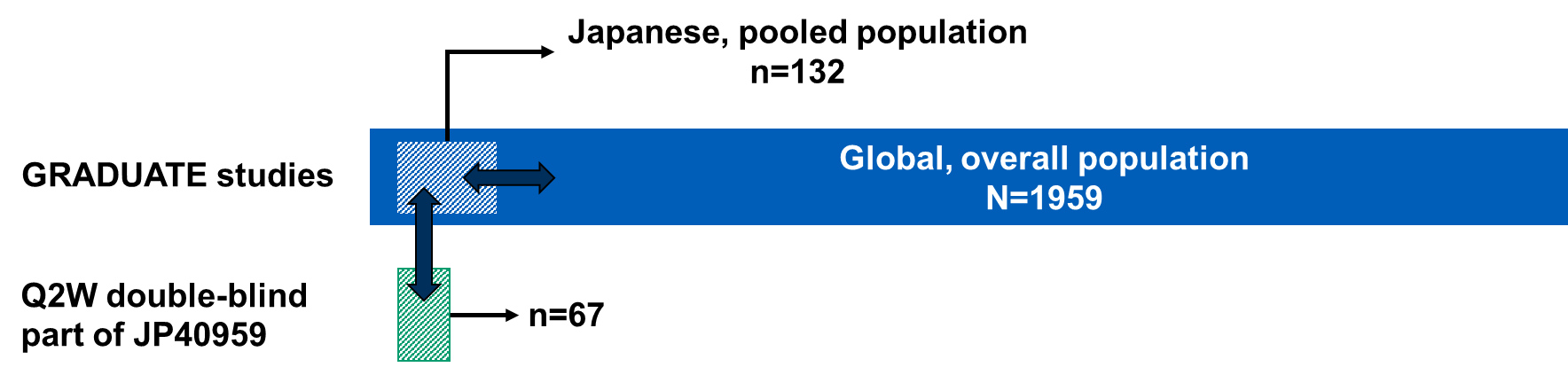


NOTE. n/N is the number of participants that were enrolled and received at least one dose of the study drug. The arrows represent comparisons between trial populations. The length of the horizontal bar represents the number of participants in the trial population.

## **SUPPLEMENTARY FIGURE 2** Study design of the Q1W open-label part of JP40959


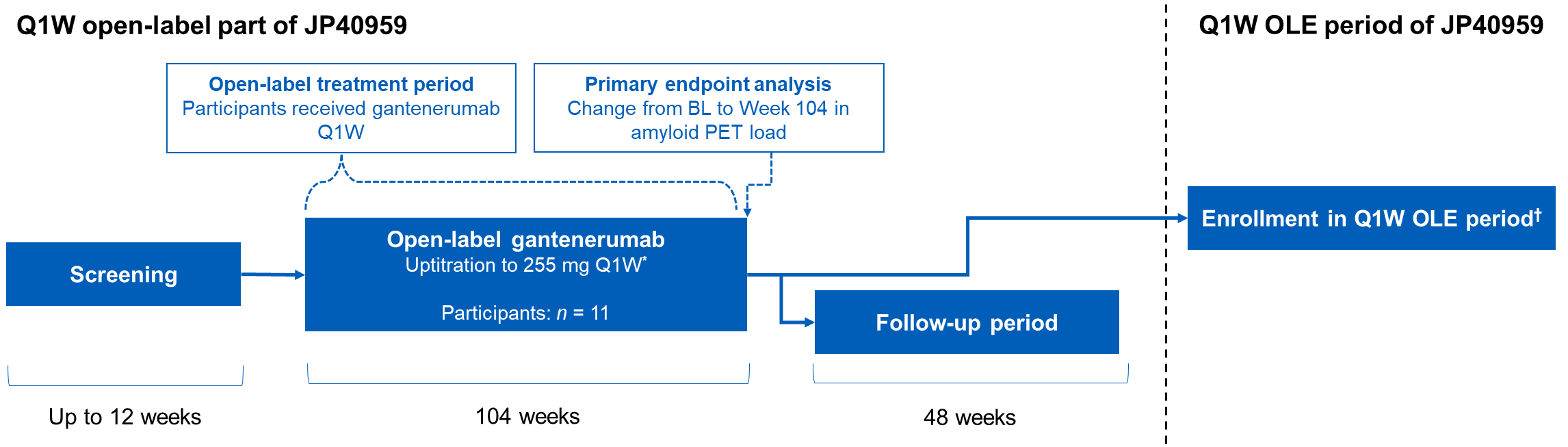


^#^Participants were eligible for the Q1W OLE periods if they provided consent to participate in the respective OLE period, had not discontinued study treatment during the initial Q1W open-label parts, and after the resolution of any ARIA MRI findings detected at week 104.

^Δ^Participants received three doses of gantenerumab 120 mg Q4W, three doses of 255 mg Q4W, six doses of 255 mg Q2W, 255 mg Q1W from week 36 onwards.

## **SUPPLEMENTARY FIGURE 3** Participant disposition of the Japanese participants in the GRADUATE studies (A), Q2W double-blind part and Q2W OLE period (B), Q1W open label (C) of JP40959

### (A) Participant disposition of the Japanese participants in the GRADUATE studies


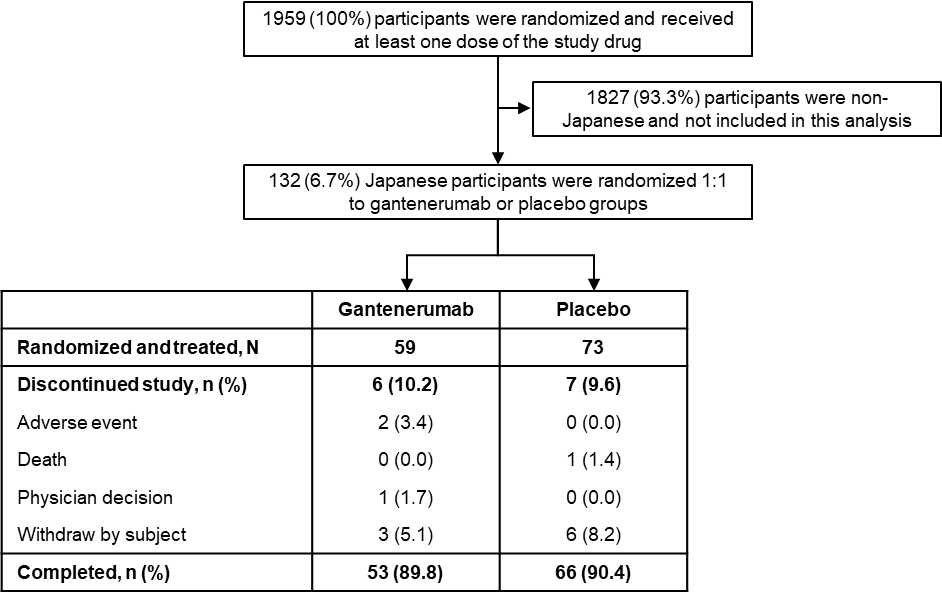


### (B) Participant disposition of the Q2W double-blind part and Q2W OLE period of JP40959


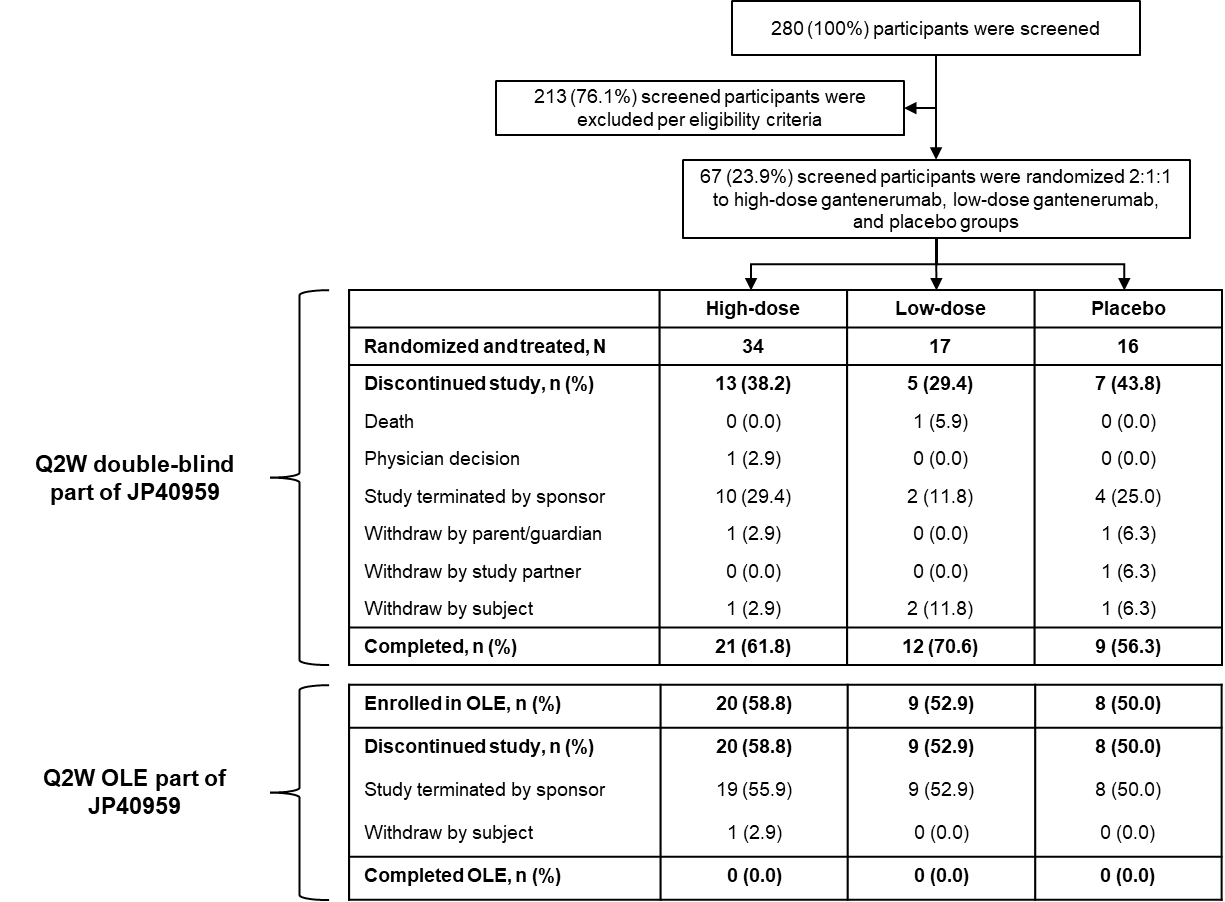


### (C) Participant disposition of the Q1W open-label part of JP40959


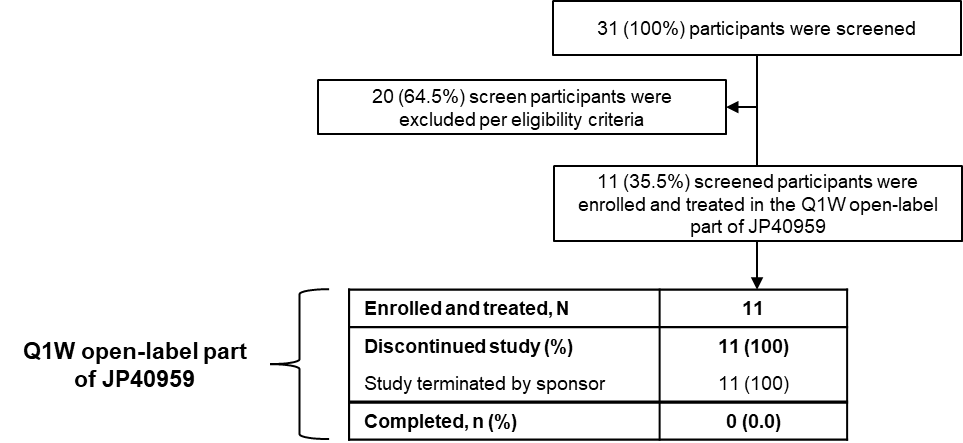


NOTE. N is the number of participants that were enrolled and received at least one dose of the study drug. JP40959 was terminated before any participants entered the Q1W OLE period.

OLE, open-label extension; Q1W, every week; Q2W, every 2 weeks.

## **SUPPLEMENTARY FIGURE 4** Adjusted mean change from baseline in cognitive and functional endpoints in the Q2W double-blind part of JP40959, and the Japanese and global populations in the GRADUATE studies

### (A) CDR-SB


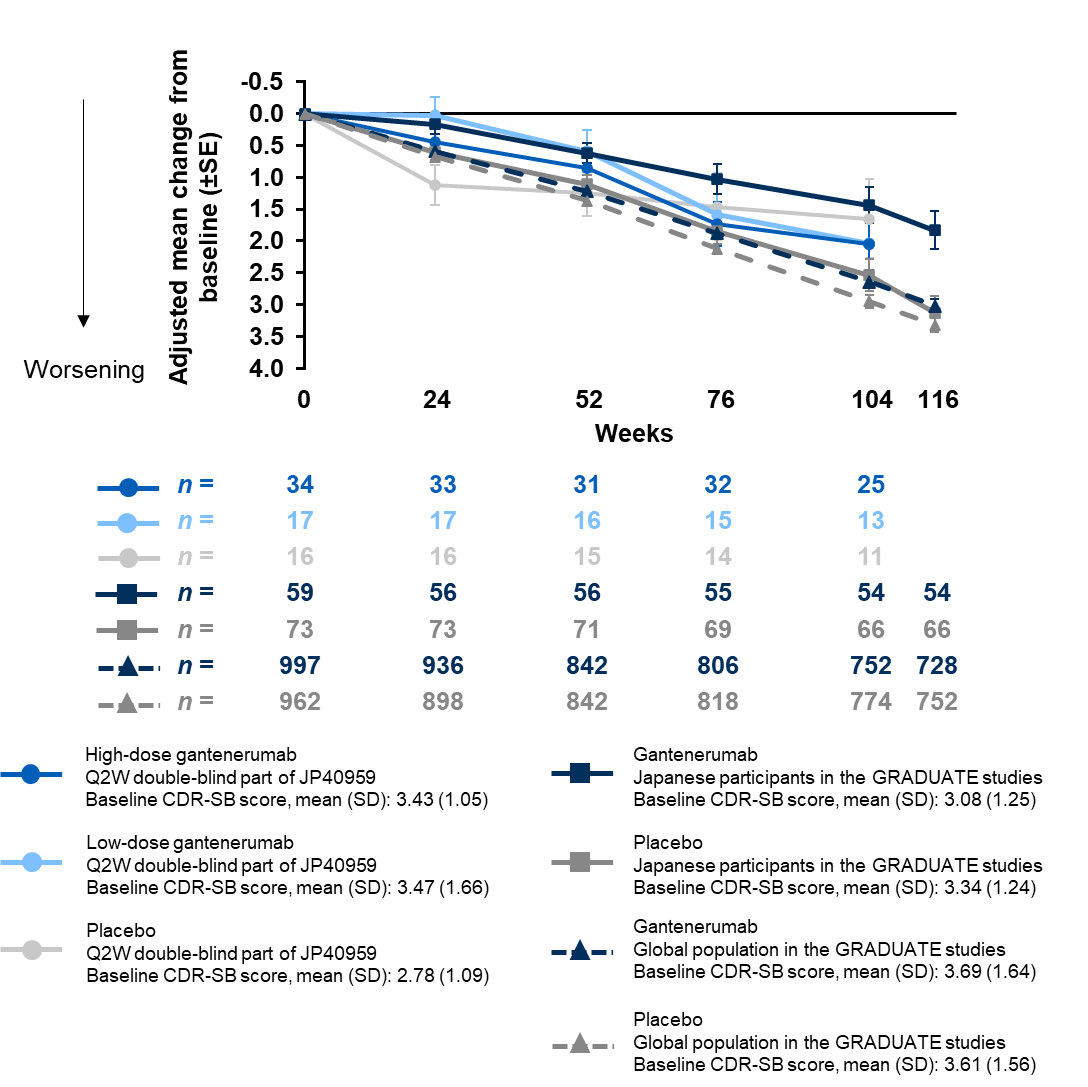


### (B) ADAS-Cog13


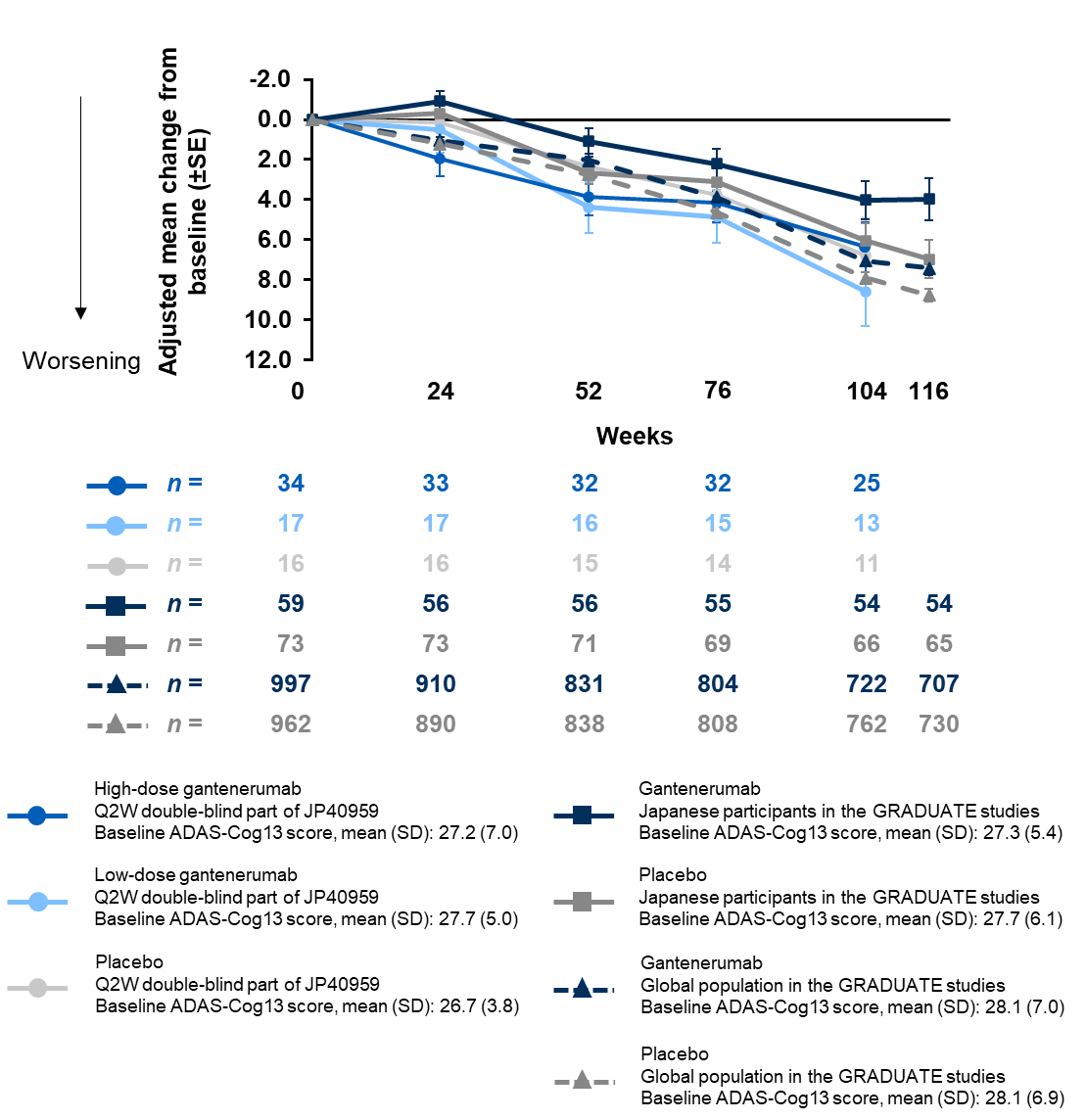


### (C) ADCS-ADL total score


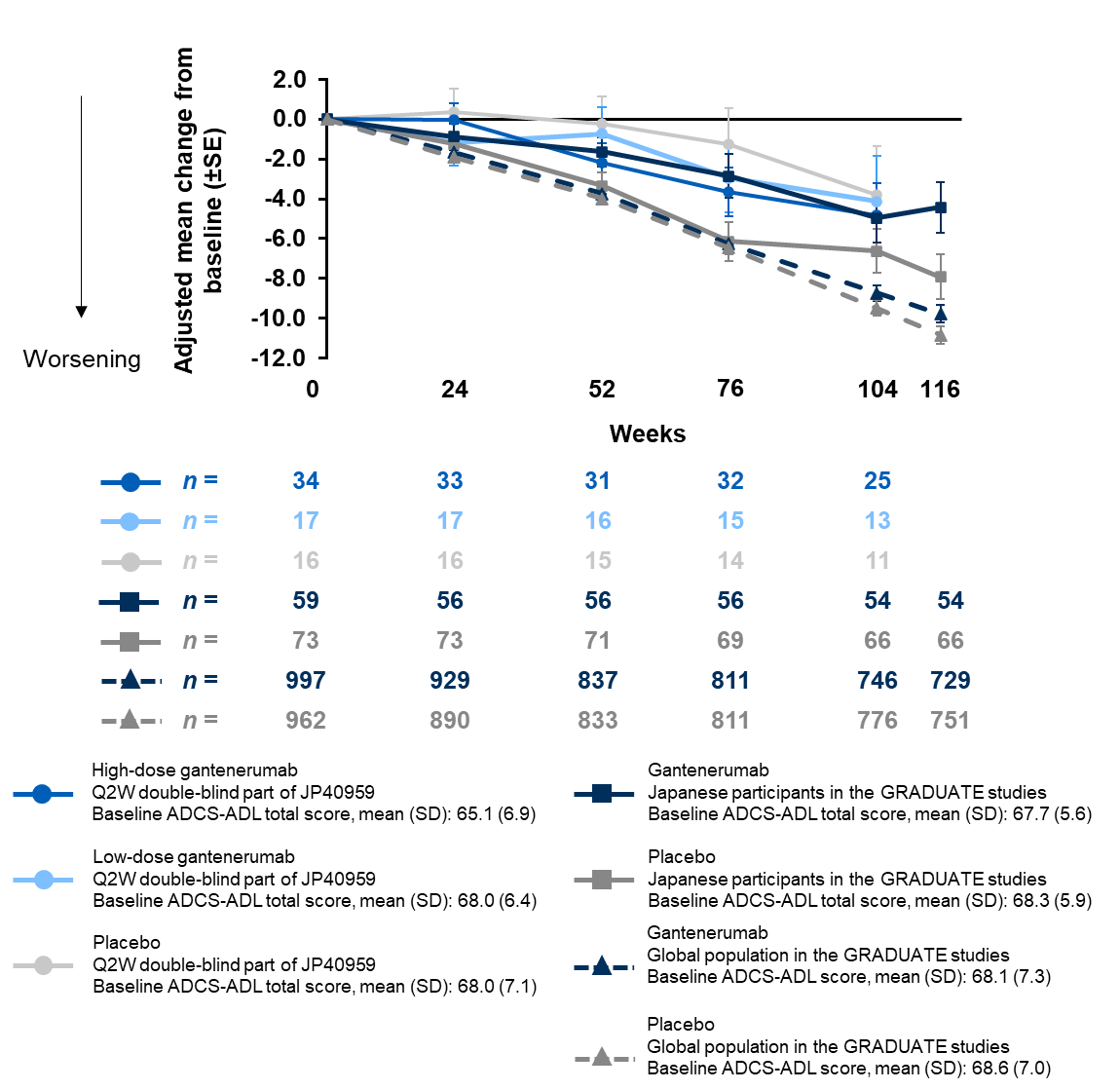


### (D) MMSE


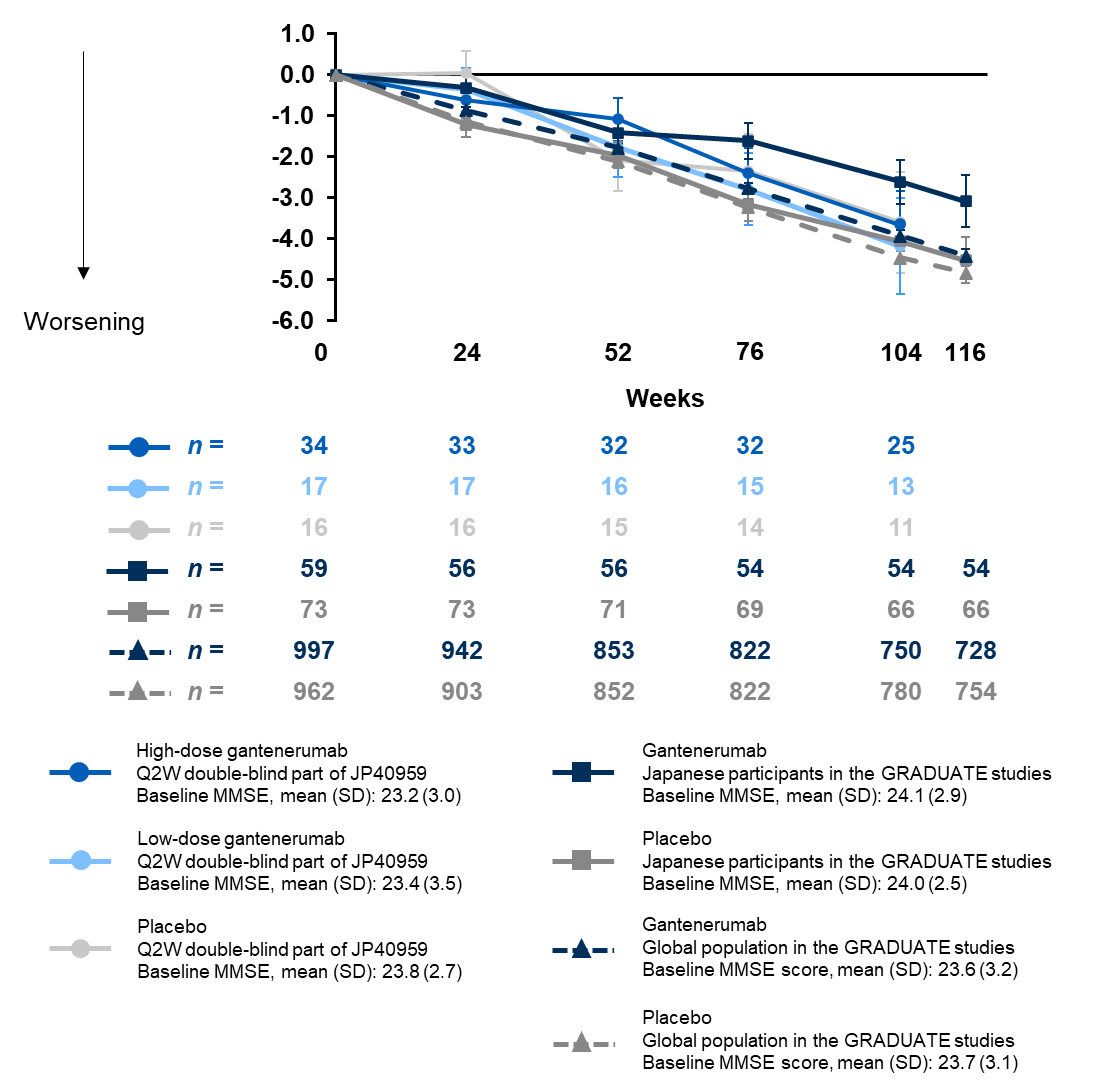


NOTE. In JP40959, change from baseline in CDR-SB, ADAS-Cog13, ADCS-ADL total score, and MMSE were assessed using an MMRM model with fixed covariate of baseline endpoint value, APOE ε4 carrier status (carrier or non-carrier), AD medication at baseline, visit, treatment group, and treatment group by visit interaction. In the global population of the GRADUATE studies, these clinical endpoints were assessed using an MMRM model; the details of the model have been reported previously [17]. For Japanese participants in the GRADUATE studies, the MMRM model excluded the geographic region as a fixed covariate that was used for the global population.

Abbreviations: ADAS-Cog13, Alzheimer’s Disease Assessment Scale – Cognitive Subscale 13; ADCS-ADL, Alzheimer’s Disease Cooperative Study – Activities of Daily Living; CDR-SB, Clinical Dementia Rating – Sum of Boxes; MMRM, Mixed Models for Repeated Measures; MMSE, Mini-Mental State Examination; Q2W, every two weeks; SD, standard deviation; SE, standard error.

## **SUPPLEMENTARY FIGURE 5** Adjusted mean change from baseline in amyloid load as measured by PET in the Q2W double-blind part of JP40959, and the Japanese and global populations in the GRADUATE studies


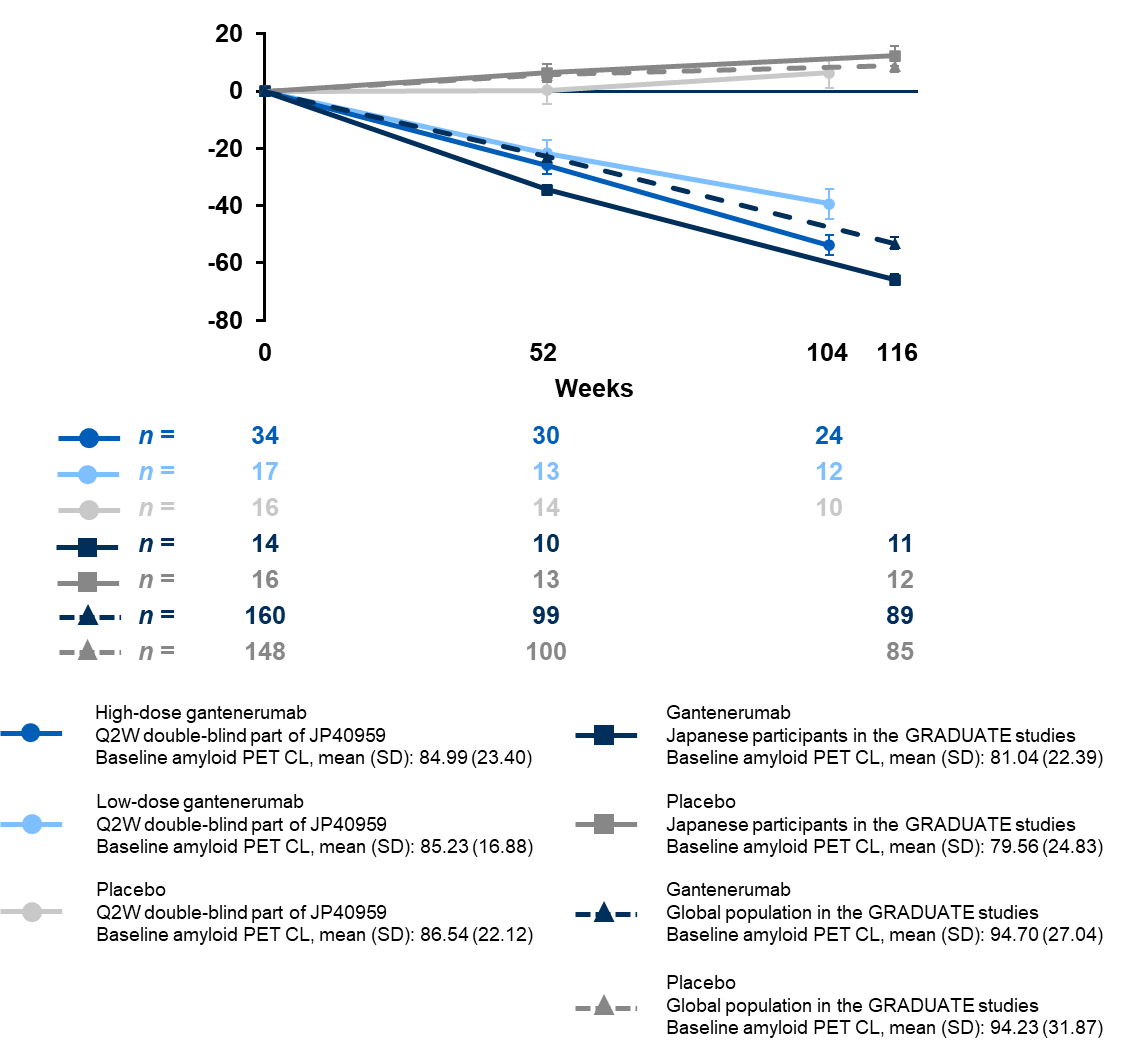


NOTE. The change from baseline in amyloid load was assessed using an MMRM model with fixed covariates of baseline amyloid load value, *APOE* ε4 carrier status (carrier or non-carrier), visit, treatment group, and treatment group by visit interaction. For global population of the GRADUATE studies the change from baseline in amyloid load were assessed using an MMRM model; the details of the model have been reported previously [17]. For Japanese participants in GRADUATE studies, the MMRM model excluded type of tracer as a covariate that was used for global population.

Abbreviations: CL, Centiloid; MMRM, Mixed Models for Repeated Measures; PET, positron emission tomography; Q2W, every two weeks; SD, standard deviation; SE, standard error.

## **SUPPLEMENTARY FIGURE 6** Mean ± SD measured values of amyloid load on PET (CL) in participants who received gantenerumab in the Japanese participants in the GRADUATE studies and JP40959

**
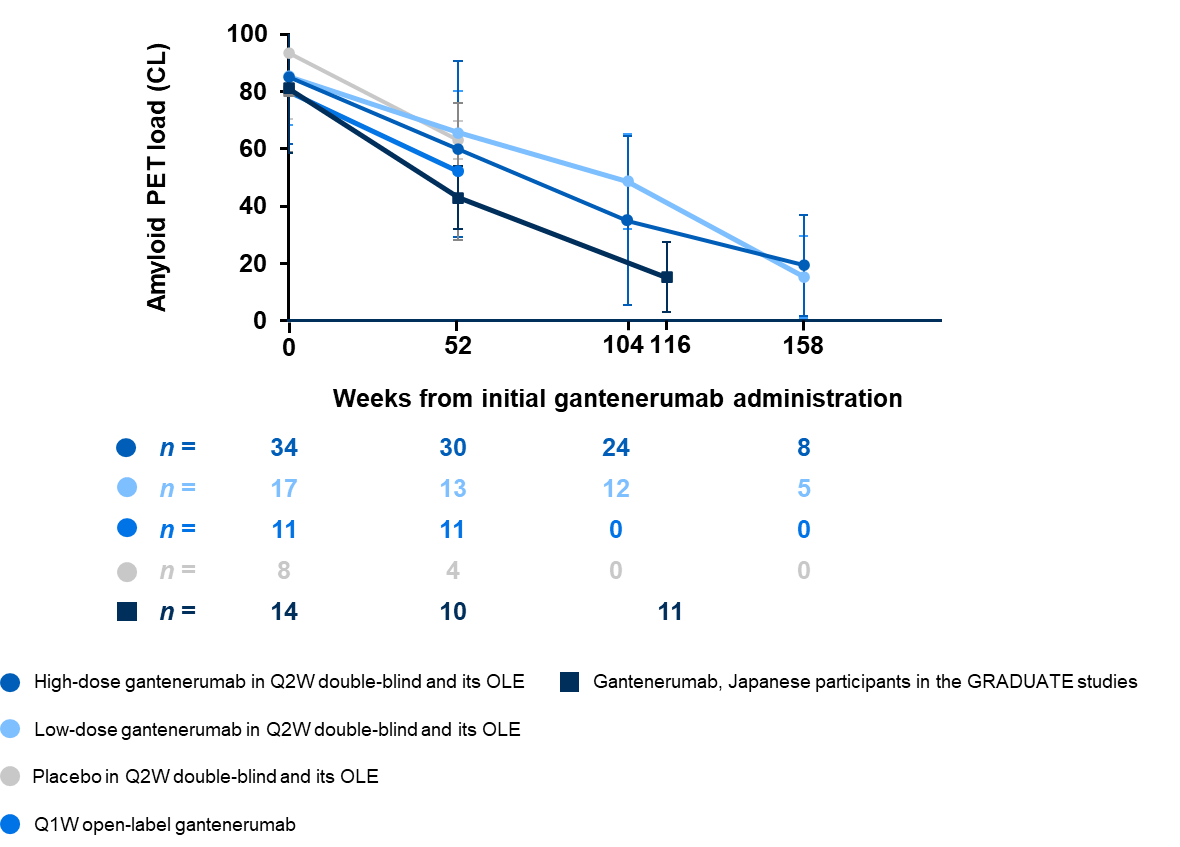
**

CL, Centiloid; OLE, open-label extension; PET, positron emission tomography; Q1W, every week; Q2W, every 2 weeks; SD, standard deviation.
